# Host-interferon-stimulated gene response to virus-host recombinant variants of hepatitis E virus and enhanced viral replication

**DOI:** 10.1128/spectrum.00600-26

**Published:** 2026-07-13

**Authors:** Olivia Paronetto, Claire Allioux, Margot Zahm, Anne Alicia Gonzalez, Nicolas Jeanne, Adeline Chaubet, Nived Collercandy, Florence Abravanel, Émeline Lhuillier, Sabine Chapuy-Regaud, Jacques Izopet, Sébastien Lhomme

**Affiliations:** 1 Institut Toulousain des Maladies Infectieuses et Inflammatoires (Infinity), UMR 5051 (CNRS), UMR 1291 (INSERM), Université́ de Toulouse, Toulouse, France; 2 Laboratoire de Virologie, Hôpital Purpan, CHU Toulouse 54904, Toulouse, France; 3 Service des maladies infectieuses et tropicales, Hôpital Purpan, CHU Toulouse, Toulouse, France; 4 I2MC, INSERM UMR 1297, Toulouse, France; 5 GeT-Santé, Plateforme Génome et Transcriptome, GenoToul 54841 , Toulouse, France; Penn State College of Medicine, Hershey, Pennsylvania, USA

**Keywords:** hepatitis E virus, virus-host recombinant variants, RNA sequencing, interferon stimulated gene, transcriptomic

## Abstract

The hepatitis E virus (HEV) is a leading cause of acute hepatitis worldwide. HEV infection can become chronic in immunocompromised individuals, in whom virus–host recombinant variants (VHRVs) can be detected. These variants often harbor host-derived insertions in the polyproline-rich region (PPR), and most display enhanced replication *in vitro*. However, the mechanisms underlying this replicative advantage remain unclear. It is likely that genes of the infected cells are differentially expressed according to the replicative capacity of the strain. The host factors involved in the improvement of the replicative capacity of these VHRVs are yet to be identified.

In this study, we analyzed the host transcriptional response to seven VHRVs in HepG2/C3A cells using bulk RNA sequencing at 48 h and 168 h post-infection. Five VHRVs (RNF19A, ZNF787, KIF1B, RPS17, EEF1A1) previously associated with a high replication rate induced more significant, distinct transcriptomic changes than low-replicative variants (RNA18, RPL6), particularly at 168 h. A shared set of 25 genes, especially interferon-stimulated genes (ISGs), was upregulated in cells infected with high-replicating variants. Interestingly, ISG induction was limited at 48 h despite high viral RNA concentrations, suggesting a delayed antiviral response. At 168 h, high ISG expression coincided with high viral loads, indicating that VHRVs may evade or exploit immune defenses. Our findings reveal candidate ISGs such as IFIT1 and ISG15 that may influence HEV persistence and immune escape. These results offer new insights into the interplay between VHRV replication and host immunity.

**IMPORTANCE:**

Hepatitis E virus (HEV) is a major cause of acute hepatitis and can cause chronic infections in immunocompromised individuals. Virus–host recombinant variants (VHRVs) having integrated host-derived insertions often replicate more effectively, yet the host determinants of this phenotype remain unclear. With RNA sequencing of HepG2/C3A-infected cells, we observed that high-replicating VHRVs induce a delayed but strong expression of interferon-stimulated genes (ISGs), including IFIT1 and ISG15, despite high viral loads. These results suggest that VHRVs may transiently modulate or evade aspects of host antiviral defenses. Our study revealed host transcriptional patterns associated with enhanced viral replication, providing insight into potential mechanisms that enhance HEV replication and highlighting candidate pathways that could influence the interplay between viral replication and immune responses, all requiring further investigation.

## INTRODUCTION

Hepatitis E virus (HEV) is a leading cause of viral hepatitis worldwide. While most infections are asymptomatic and self-limiting, severe outcomes such as acute liver failure can occur, particularly in pregnant women and individuals with pre-existing liver disease (1). HEV has also been associated with extrahepatic manifestations, including neurological and renal disorders (2). In immunocompromised patients, HEV disease may progress to chronic infection, defined by viral persistence beyond three months (3) and may potentially lead to accelerated liver fibrosis (4, 5). Ribavirin monotherapy for 3 months is currently the recommended treatment for chronic HEV infection (6) due to its proven efficacy (7).

HEV belongs to the *Hepeviridae* family. Most human cases are due to strains of the *Paslahepevirus balayani* species*,* with four main genotypes (8). HEV1 and HEV2 are human-restricted, while HEV3 and HEV4 are zoonotic, with pigs and wild animals as reservoirs (8). HEV is a quasi-enveloped virus: particles released into the blood and cell culture supernatant are membrane-associated, whereas particles in feces are naked (9).

HEV is a positive-sense, single-stranded RNA virus of approximately 7.2 kb, comprising three main open reading frames (ORFs). ORF1 encodes the nonstructural polyprotein essential for replication, including a methyltransferase, macrodomain, helicase, and an RNA-dependent RNA polymerase, as well as a less characterized domain named the polyproline region (PPR). ORF2 encodes the capsid protein, and ORF3 encodes a multifunctional protein involved in viral egress (10).

Recombinant HEV3 variants containing insertions of human gene fragments or viral genome duplications in the PPR have been described in immunocompromised patients. The Kernow p6 strain, which harbors a fragment of the human ribosomal gene S17, is the most-characterized recombinant HEV variant and shows increased replication compared to its parental strain which lacks this insertion (11, 12). Similarly, strains LBPR-0379 (with a fragment derived from the S19 human gene) and TLS-09 (with a fragment derived from the ITI-H2 human gene) (13, 14) also display a replicative advantage *in vitro*. We previously demonstrated that five additional virus-host recombinant variants (VHRVs), namely RNF19A, ZNF787, KIF1B, RPS17, and EEF1A1, have a replicative advantage *in vitro*, while two others, RNA18 and RPL6, did not (15). All these VHRV strains were HEV3f except RNF19A (HEV3h) and RPL6 (HEV3m). To date, the mechanisms underlying this replicative advantage remain poorly understood.

Interferons (IFNs) play a key role in innate immunity, triggering the expression of hundreds of interferon-stimulated genes (ISGs) that act through various mechanisms to limit viral replication (16). During HEV infection, type III IFNs (IFN-⌊) are predominantly induced, particularly following genotype 3 (Kernow p6) infection in the hepatoma cell line HepG2/C3A and primary human hepatocytes (17). Although ISG induction has been widely reported *in vitro* and *in vivo* (17–19), HEV can still replicate in such an antiviral environment. HEV employs multiple strategies to escape innate immunity. Viral proteins encoded by ORF1, ORF2, and ORF3 interfere with innate immune signaling by targeting key components of IFN pathways, including RIG-I, TBK1, IRF3, NF-|B, and JAK/STAT (20–24). HEV also modulates the expression of ISGs such as IFIT1 and ISG15, which play opposite roles in the viral life cycle. IFIT1 exerts an antiviral effect by targeting viral RNA of HEV1 (25), while ISG15 has been shown to promote HEV3 replication through a JAK/STAT-dependent mechanism involving ISGylation (26), the covalent conjugation of ISG15 to target proteins (27). These immune evasion strategies could help viral replication, although the mechanisms involved are not fully understood. It is possible that the profiles of induced ISGs differ depending on whether the strain used replicates well.

To date, transcriptomic analysis of recombinant HEV infection has only been conducted using the strain Kernow p6 (18), and no comparison with non-recombinant strains has ever been performed. Determining which genes, especially ISGs, are differentially expressed during infection would be helpful to identify potential host factors involved in the improvement of the replication capacity of VHRVs.

In this study, we performed a global transcriptomic analysis of HepG2/C3A cells infected with the seven newly described VHRVs and focused on ISGs expression to better understand the host factors associated with their replication phenotypes.

## RESULTS

**Fig 1:**
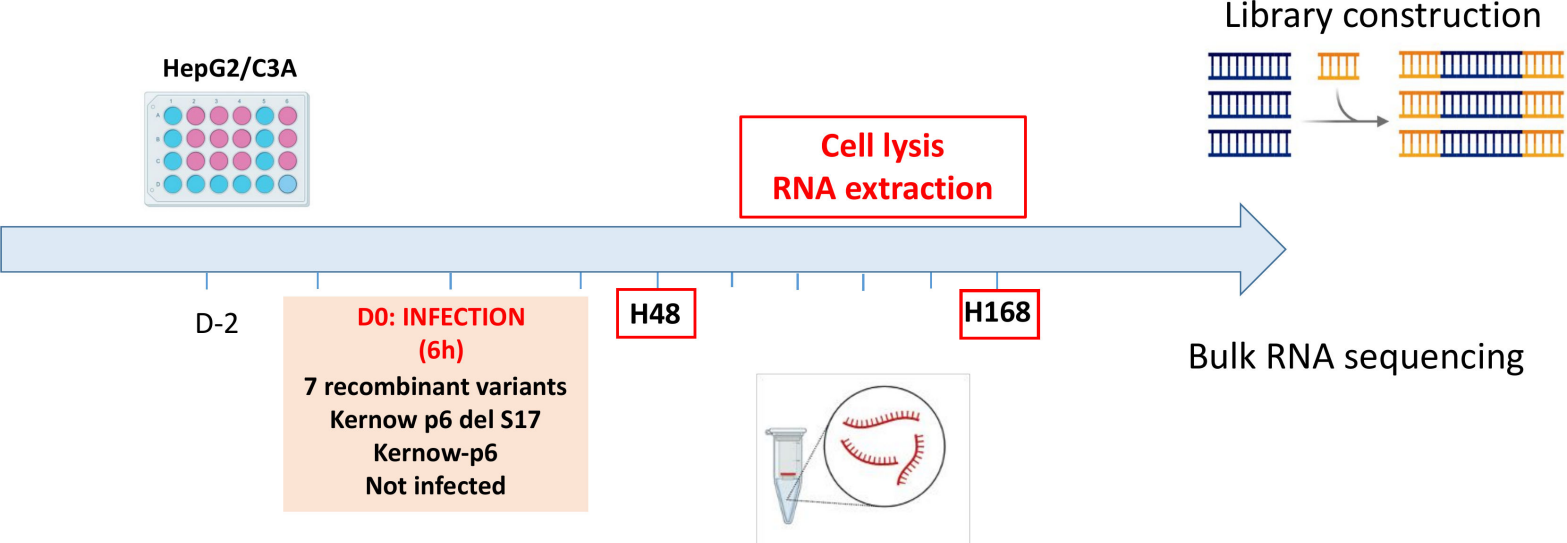
Experimental design for RNA-seq analysis of HepG2/C3A cells infected with each of the VHRVs. HepG2/C3A cells were seeded in 24-cell plates and infected after 24 h (day 0) with each of the seven VHRVs, the strain without the S17 insertion (Kernow del S17), or left uninfected. Infection was performed 6 h before washing. Samples were collected at 48 h and 168 h post-infection. At each time point, cell lysates were harvested for RNA extraction and transcriptomic profiling by bulk RNA sequencing. For each condition and time point, three independent biological replicates were collected. Each tick denotes an interval time of 24 h.

To study host transcriptional response to VHRVs, we performed bulk RNA sequencing on HepG2/C3A cells infected with the seven previously characterized VHRVs (28). The strain without the S17 insertion (Kernow del S17), Kernow p6, and non-infected cells were used as controls. Infections were carried out for 6 h, after which cells were washed three times and incubated with fresh medium. Cell lysates were collected at 48 h and 168 h post-infection to capture both early and late stages of host response, as performed previously by Todt et al. (18) who described the highest upregulation of IFN-regulated genes after 48 h. The 168 h post-infection time point was tested due to the low replicative rate of some strains to investigate a possible delayed upregulation of ISGs. Total RNA was extracted from cell lysates and subjected to transcriptomic profiling. The experimental setup is summarized in Fig. 1.

### Principal component analysis (PCA) of host genes differentially expressed in response to VHRV infection

**Fig 2:**
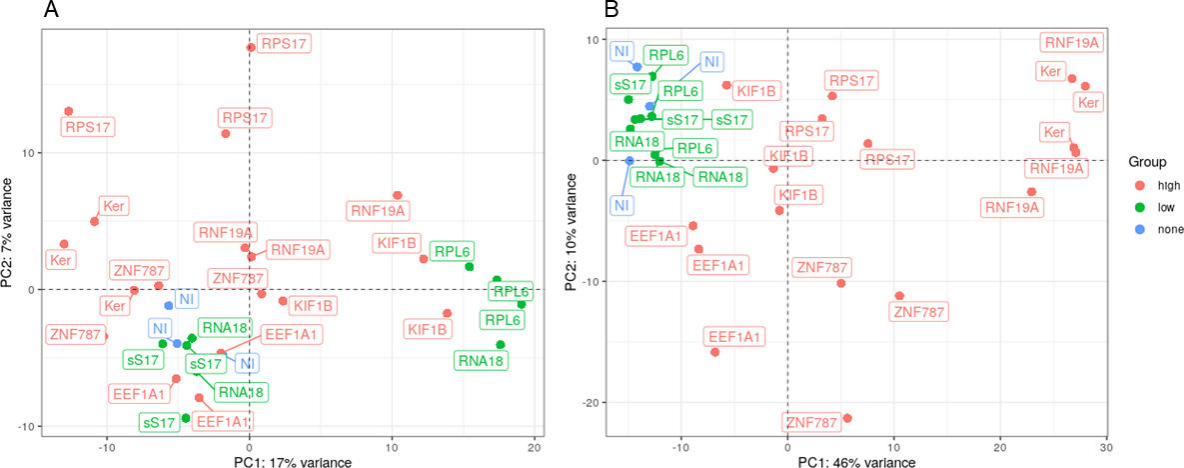
Score plot illustrating PCA spectral analysis for DEGs between the VHRV at 48 h (**A**) and 168 h (**B**). The VHRVs with a replicative advantage (Kernow p6, RNF19A, ZNF787, RPS17, KIF1B, EEF1A1) are presented in pink. The viruses without a replicative advantage (RPL6 and RNA18) and the strain without insertion (named sS17) are in green. The non-infected condition is shown in blue.

To visualize the variation in gene expression profiles in the cells infected by the different VHRVs at 48 h and 168 h post-infection, we performed a PCA on the set of differentially expressed genes (DEGs) (Fig. 2). PCA was performed on the normalized expression values of the top 500 most variable genes. This method projects the high-dimensional gene expression data onto a two-dimensional space defined by the first two principal components, allowing perception of the main differences between VHRVs at the two time points post-infection (29). Therefore, the principal component with the highest variance in gene expression profile would be the best feature that would allow us to separate the VHRVs with or without a replicative advantage. The addition of PC1 and PC2 for each time point allows us to calculate the total variance (29). We observed that PC1 and PC2 explained 24% and 56% of the total variance, at 48 h and 168 h post-infection, respectively. At 48 h post-infection, the PCA analysis revealed that RNA18, the strain without the insertion, and the non-infected condition clustered closely together. Only one RNA18 replicate appeared isolated from the main cluster. In contrast, the other VHRVs showed a more dispersed distribution across the PCA space (Fig. 2A). At 168 h post-infection, the projection revealed distinct clustering patterns according to the VHRV. The VHRVs without replicative advantage formed a clear separate cluster with the non-infected condition and the strain without the insertion. RNF19A and Kernow p6 were positioned close together, similarly to RPS17 and KIF1B. Unlike the other strains with a replicative advantage, EEF1A1 and ZNF787 showed a broader distribution among the principal components at 168 h post-infection (Fig. 2B).

### Gene Ontology (GO) enrichment analyses of biological processes in response to VHRVs infection

**Fig 3:**
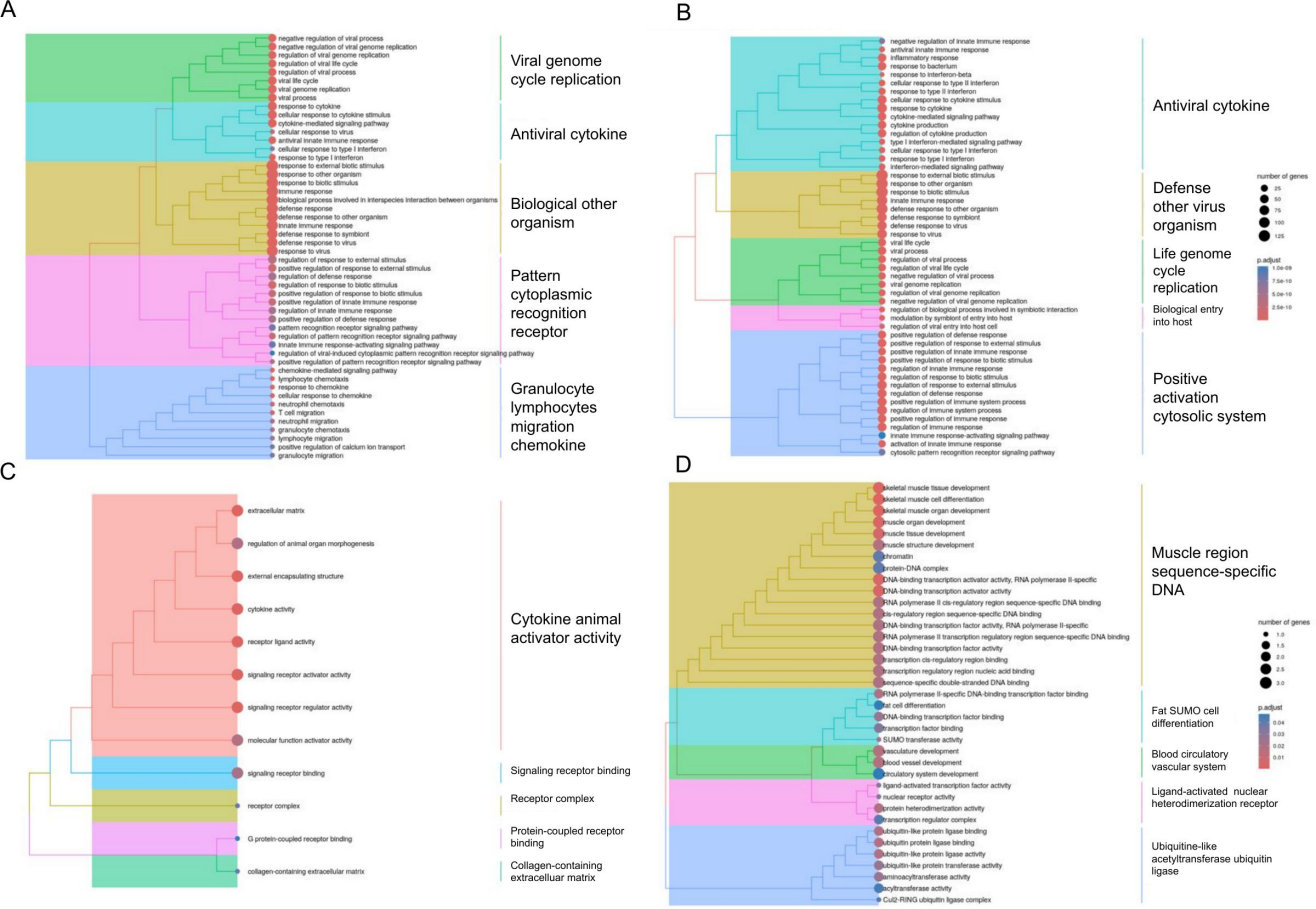
Gene Ontology enrichment analysis of differentially expressed genes in HepG2/C3A cells infected with RNF19A or RPL6. (**A and B**) Infection of cells with RNF19A at 48 h (**A**) and 168 h (**B**) post-infection. (**B and C**) Infection of cells with RPL6 at 48 h (**C**) and 168 h (**D**) post-infection. Analyses were performed using clusterProfiler (v.4.10.0) based on the GO Biological Process database.

At 48 h post-infection, cells infected with strains having a high replicative rate, such as RNF19A (Fig. 3A), RPS17 (Fig. S1A), ZNF787 (Fig. S2A), and Kernow p6 (Fig. S3A), exhibited GO enrichment related to viral replication and life cycle processes. Enriched terms included “viral genome replication,” “viral life cycle,” and “regulation of viral genome replication,” involving 3–15 genes per term. Despite the relatively modest gene counts, enrichment was statistically significant (adjusted *P* values < 0.0025). In addition, early activation of innate immunity was observed, with terms such as “antiviral innate immune response” and “cellular response to cytokine stimulus.” These pathways were not shared with EEF1A1 and KIF1B at the early stage of the infection (Fig. S4A) but only at 168 h post-infection (Fig. S4B and S5). In contrast, at 48 h post-infection, cells infected with low replicative rate variants demonstrated enrichment of innate immune-related pathways, such as “Toll-like receptor signaling” and “receptor internalization.” In RPL6-infected cells, GO enrichment analysis at 48 h revealed predominant modulation of signaling and extracellular structure-related pathways, including terms such as “cytokine activity,” “receptor ligand activity,” and “signaling receptor activator activity” (Fig. 3C).

At 168 h post-infection, the transcriptional landscape was associated with high activation of immune-related pathways for RNF19A (Fig. 3B), RPS17 (Fig. S1B), ZNF787 (Fig. S2B), and Kernow p6 (Fig. S3B). Enriched GO terms included “interferon-mediated signaling pathway,” “regulation of immune response,” and “positive regulation of innate immune response.” These pathways were associated with a markedly higher number of DEGs (often 50–100 genes per term) and adjusted *P* values < 1.0 × 10⁻⁹ (Fig. 3B). Several terms related to immune amplification, including “type I/II interferon responses,” “cytokine-mediated signaling pathway,” and “pattern recognition receptor signaling,” were also significantly enriched.

Additionally, the pathways related to immune cell migration and chemotaxis, including “neutrophil chemotaxis” and “granulocyte migration,” were also enriched at 168 h post-infection, notably in RNF19A (Fig. 3B), RPS17 (Fig. S1B), and Kernow p6 (Fig. S3B).

In RPL6-infected cells, GO enrichment at 168 h indicated a transition toward transcriptional regulation and protein modification, with terms such as “transcription regulator complex,” “DNA-binding transcription factor activity,” “ubiquitin-like protein ligase activity,” and “SUMO transferase activity.” Terms related to “skeletal muscle tissue development” and “vasculature development” were also enriched (Fig. 3D). RNA18-infected cells showed a transcriptional profile similar to RPL6, with enriched terms associated with transcriptional regulation and DNA binding. However, RNA18 exhibited a stronger emphasis on “skeletal muscle differentiation” and “hormone-mediated signaling” and lacked enrichment in post-translational modification pathways, such as ubiquitination or SUMOylation (Fig. S6).

### Intersection analysis of up- and downregulated genes at 168 h post-infection

**Fig 4:**
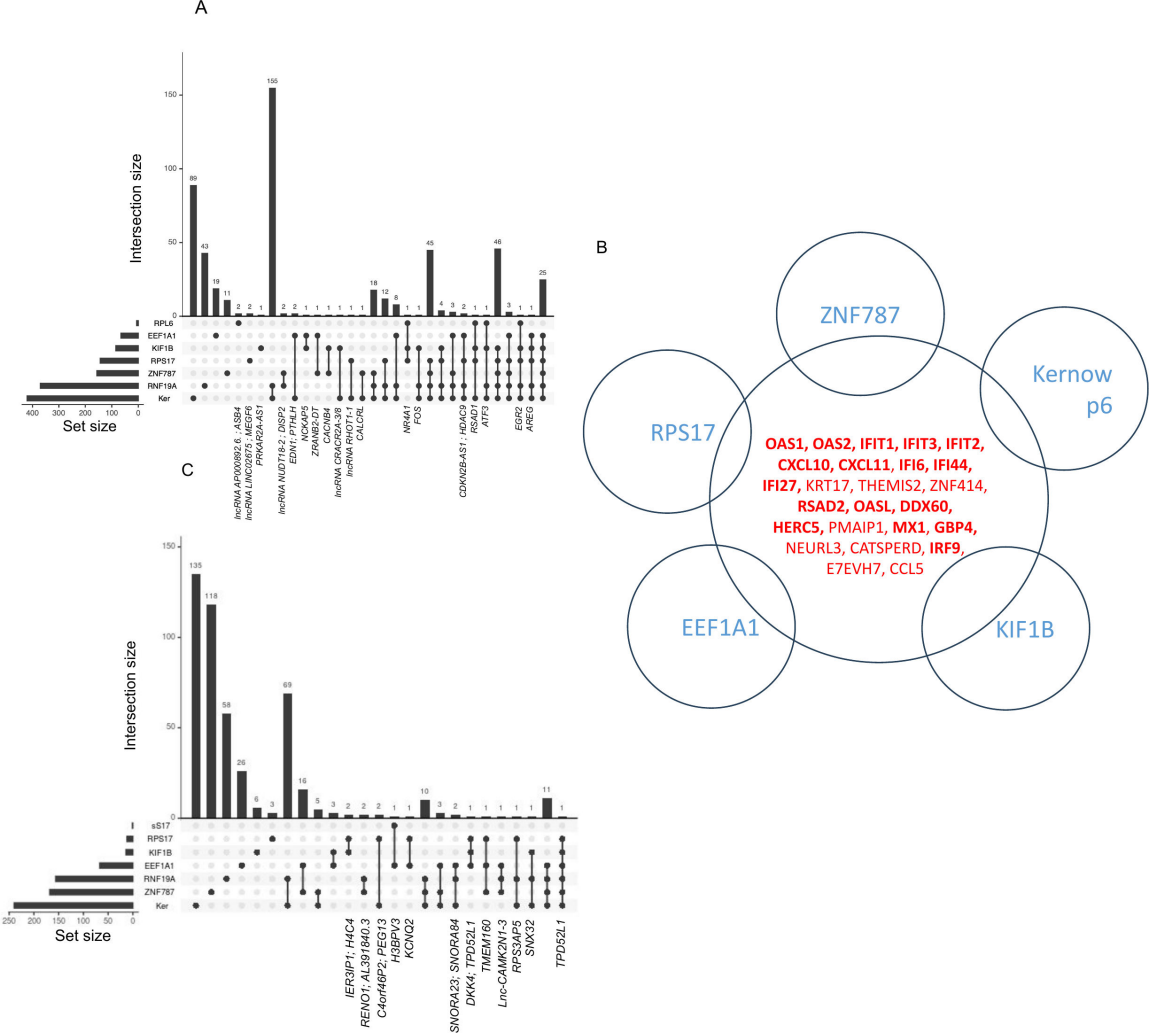
UpSet plots showing the number of differentially expressed genes shared among VHRVs at 168 h post-infection. Representation of upregulated genes (**A**) common upregulated genes for the VHRVs with a high replication rate (**B**) and downregulated genes (**C**). Rows represent sets, and columns represent their intersections. Filled circles indicate set membership, and lines connect them to show the intersection pattern. Intersection sizes are shown as bars above the matrix, and set sizes are shown as bars next to each row. Genes in bold red correspond to ISGs.

We performed a cross-strain analysis of DEGs to identify common and distinct sets of upregulated genes, reflecting both shared and strain-specific transcriptional responses at 168 h post-infection (Fig. 4A). Only VHRVs with at least one significantly upregulated gene are shown. VHRVs without differentially expressed genes at the selected threshold Kernow p6 del S17 and RNA18 are not presented. The infection of HepG2/C3A cells with Kernow p6 and RNF19A induced, respectively, the upregulation of 89 (such as *NFKBIA* encoding the IκBα protein) (Table S1) and 43 genes (such as *STAT2*) (Table S2; Table S2). Totals of 19 and 11 were, respectively, expressed after the infection with the recombinants EEF1A1 (such as *NGFR* and *TNFRSF11B*, respectively, encoding receptors and cytokines of the TNF family) (Table S3) and ZNF787 (Table S4). The strains RNF19A and Kernow p6 shared the largest number of genes (155 genes, including *Myd88* and *IRF1*) listed in Table S5. RPS17, RNF19A, and Kernow p6 shared 12 genes (Table S6), including those encoding CCL2 and granzyme A. ZNF787, RNF19A, and Kernow p6 shared 18 genes, including *TLR3* and *DDX60L* (Table S7). Eight genes were upregulated (including *CDKN2B* which encodes a cyclin-dependent kinase inhibitor) for EEF1A1, RNF19A, and Kernow p6 (Table S8). Only the group with KIF1B, RPS17, RNF19A, and Kernow p6 contained four genes: *CXCL2*, *SOCS2*, *ODF3B*, and *FCF1P2*. Three genes, *CAV1*, *TNF*, the microRNA precursor gene *MIR941-5,* and *DAPP1*, MAP2, and *ARL14* were, respectively, found for the first group EEF1A1, ZNF787, RNF19A, and Kernow p6, and the second group EEF1A1, RPS17, ZNF787, RNF19A, and Kernow p6. Interestingly, 25 genes, most being ISGs such as IFIT and OAS, were commonly upregulated for all VHRVs with a replicative advantage (Fig. 4B and Table S9). On analysis of the genes shared between five VHRVs with a replicative advantage, *AREG* was upregulated (Fig. 4A). *AREG* encodes an EGFR ligand implicated in tissue repair and immune regulation (30). The analysis revealed 45 genes associated with RPS17, ZNF787, RNF19A, and Kernow p6 (such as *IFI35*, *MX2*), while the inclusion of KIF1B in the VHRVs group led to the identification of 46 genes. The full list of commonly upregulated genes is provided in Table S10.

We also examined the overlap of significantly downregulated genes across VHRV strains at 168 h post-infection (Fig. 4C). The extent of gene repression varied substantially between strains. Kernow p6, ZNF787, and RNF19A exhibited the largest sets of uniquely downregulated genes, with 135, 118, and 58 genes, respectively (listed in Tables S11–S13). Smaller gene sets were observed for EEF1A1 (26 genes shown in Table S14), five genes for KIF1B (such as *EIF4EBP3* involved in “viral genome replication” and “viral life cycle”), and three genes for RPS17 (*PLP1*, *ZNF436-AS1*, and 1 gene with no assigned name). The highest overlap was observed between RNF19A and Kernow p6 with 69 shared downregulated genes (Table S15). EEF1A1 and ZNF787 shared 16 genes (Table S16), while RNF19A, ZNF787, and Kernow p6 shared 10 genes. EEF1A1, RNF19A, ZNF787, and Kernow p6 commonly downregulated these 11 genes: *SNORA52*, *SNORA71D*, *SNORA38*, *RNY1*, *RNY3*, *RNU4-2*, *SNORA71A*, *SNORD13*, *SNORA67*, *H4C1,* and one gene with no assigned name. ZNF787 and Kernow p6 also shared the following genes: *RNU5A-1*, *RNU4-1*, *SNORA3A*, *SNORA77B*, and *H4C4*. The groups KIF1B, ZNF787, Kernow p6, and EEF1A1, respectively, shared three downregulated genes: *PLPPR1*, *SNORA80B*, *SNORA8*, *SLC22A7*, and *ALDH3A1* related to “cytokine-mediated signaling pathway” and *ETNPPL* related to “hormone-mediated signaling.” Only two genes were shared for four distinct groups: two genes with no assigned name for RPS17 and KIF1B; *RENO1* and one gene with no assigned name for RNF19A and ZNF787; *C4orf46P2* and one gene with no assigned name for RPS17, Kernow p6, and *SNORA23*; and *SNORA84* for EEF1A1, RNF19A, and Kernow p6. The other groups shared one gene: a gene with no assigned name for Kernow p6 del S17 and EEF1A1; *KCNQ2 and TMEM160* for RPS17 and EEF1A1; *DKK4* (related to “skeletal muscle tissue development” and “vasculature development”) for RPS17, KIF1B, and EEF1A1; *TMEM160* for RPS17, EEF1A1, and ZNF787; *RPS3AP5* for RPS17, RNF19A, and Kernow p6; and *SNX32* (related to “receptor internalization”) for KIF1B, RNF19A, and Kernow p6. Interestingly, a single gene *TPD52L1* (associated with “receptor internalization”) was commonly downregulated across all six VHRVs with a replicative advantage of RPS17, KIF1B, EEF1A1, RNF19A, ZNF787, and Kernow p6.

### Differentially expressed ISGs in VHRV-infected HepG2/C3A cells

**Fig 5:**
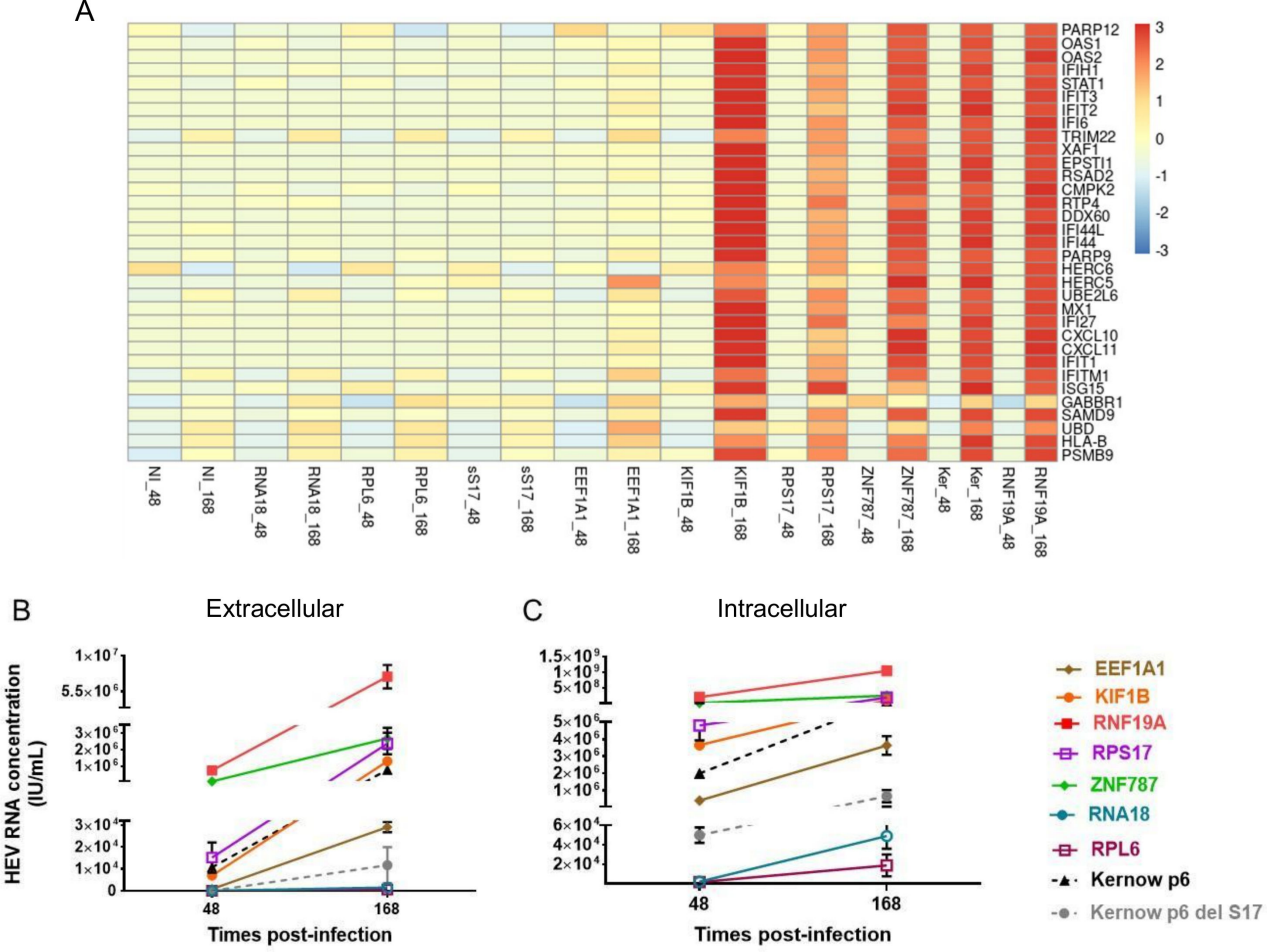
Coordinated ISG response and viral release dynamics in HepG2/C3A cells following VHRVs infection. (**A**) Heat map showing relative expression of 33 selected ISGs in cells infected with the seven VHRVs compared to the non-infected cells. (**B and C**) Kinetics of HEV RNA release in the supernatants (**B**) or in the cell lysates (**C**) of HepG2/C3A infected with each of the seven VHRVs. We used 1 × 10^7^ copies for 6 × 10^5^ cells, which corresponds to 17 GE/cell. At 48 h and 168 h post-infection, HEV RNA was extracted from the culture supernatants or cell lysates and quantified by RT-qPCR. The results show the mean ± SD of three biological replicates.

Next, we investigated whether the ISG response was modulated in HepG2/C3A cells upon infection with VHRVs at 48 h and 168 h post-infection. We selected a non-exhaustive list of 33 ISGs to perform an analysis based on previously published data (18, 31). The expression of ISGs remained largely unchanged in cells infected with the low replicative rate virus l (RNA18 and RPL6) or the strain without insertion, both at 48 h and 168 h post-infection (log2 fold changes close to zero) (Fig. 5A). The transcriptional profile was not significantly modified for the VHRVs with a replicative advantage at 48 h post-infection. In contrast, a marked upregulation of 31 ISGs was observed at 168 h post-infection for RNF19A, ZNF787, KIF1B, and Kernow p6 with fold change ranging from 2 to 3 (Fig. 5A). These ISGs are involved in diverse antiviral mechanisms, including viral RNA sensing (*IFIH1*, *DDX60*, *RTP4*), inhibition of viral translation (*IFIT1*, *IFIT2*, *IFIT3*, *IFI6*, *MX1*, *SAMD9*, *IFITM1*), RNA degradation via the OAS-RNase L pathway (OAS1, *OAS2*, *RSAD2*), and ISGylation-related processes (*ISG15*, *UBE2L6*, *HERC5*, *HERC6*). Other genes participate in IFN signaling and transcriptional regulation (*STAT1*, *PARP9*, *PARP12*, *XAF1*, *EPSTI1*, *CMPK2*) or contribute to immune modulation and antigen presentation (*CXCL10*, *CXCL11*, *HLA-B*, *PSMB9*, *TRIM22*, *IFI27*, *IFI44*, *IFI44L*). The gene *UBD* was systematically upregulated, although to a lesser extent for the same VHRVs. At 168 h post-infection, ISG induction in the cells infected with RPS17 appeared less pronounced overall, except for *ISG15*, which was strongly induced. The gene *GABBR1* encoding the GABA type B receptor subunit 1 displayed a heterogeneous expression profile across the five VHRVs. At 168 h post-infection, *GABBR1* was moderately upregulated for KIF1B, RNF19A, and Kernow p6. Its expression remained closer to baseline for RPS17 and ZNF787 (Fig. 5A). HEV RNA concentrations were measured in the supernatant and in the cell lysate at 48 h and 168 h post-infection, as shown in Fig. 5B and C, respectively. The highest number of ISGs upregulated was observed at 168 h post-infection for VHRVs with a replicative advantage, which is consistent with the high levels of viral RNA detected in both the cell lysates and supernatants.

### Validation of the RNA sequencing data by immunoblotting and ELISA

**Fig 6:**
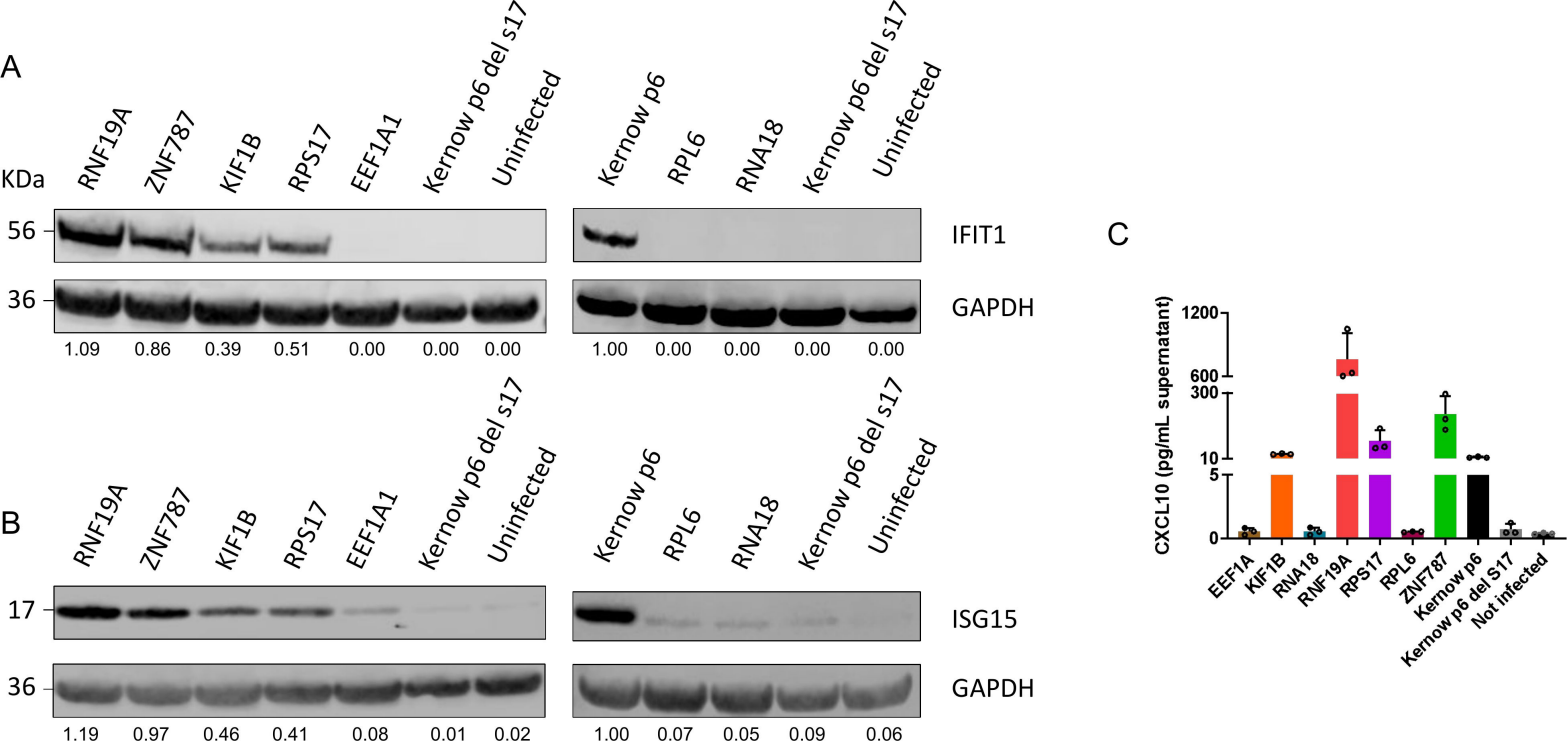
Protein-level validation of selected genes identified in transcriptomic analysis. Western blots of IFIT1 (**A**) and ISG15 (**B**) proteins in HepG2/C3A cells 168 h after infection with each of the VHRVs and Kernow p6 del S17. GAPDH served as the loading control. Band intensity was first normalized with GFP, and the ratio was compared to the positive control Kernow p6. (**C**) Detection of CXCL10 in the supernatant of non-infected HepG2/C3A or HepG2C3A infected with each of the VHRVs at 168 h post-infection by ELISA. The results shown are the means ± SD from three biological replicate samples.

To confirm the transcriptional upregulation of IFIT1 and ISG15 observed in RNA sequencing analysis, we evaluated their protein expression in the lysates of HepG2/C3A cells infected with the VHRVs by western blotting (Fig. 6A). The IFIT1 protein expression was observed only in cells infected with ZNF787, RNF19A, KIF1B, RPS17, and Kernow p6 at 168 h post-infection. In contrast, the ISG15 expression was detected in all the conditions with a low level of expression for RNA18, RPL6, and Kernow p6 del S17 (Fig. 6B). ELISA assays were also performed to quantify CXCL10 protein levels in the supernatants of HepG2/C3A cells infected with the VHRVs (Fig. 6C). A strong secretion of CXCL10 was observed for RNF19A with concentration reaching 760.1 ± 144.2 pg/mL. ZNF787, KIF1B, RPS17, and Kernow p6 led to a moderate increase (206.1 ± 46.7 pg/mL, 29.6 ± 1.2 pg/mL, 87.3 ± 28.3 pg/mL, and 15.5 ± 1.8 pg/mL, respectively). In contrast, CXCL10 concentrations remained low for cells infected with RNA18, RPL6, EEF1A1, and Kernow p6 del S17 (0.6 ± 0.2 pg/mL for RNA18, 0.5 pg/mL for RPL6, 0.6 ± 0.2 pg/mL for EEF1A1, and 0.7 ± 0.3 pg/mL for Kernow p6 del S17).

## DISCUSSION

In this study, we analyzed the host transcriptomic response to seven VHRVs of HEV in HepG2/C3A cells to identify potential host factors involved in the improvement of the replication capacity of VHRVs, with a particular focus on ISGs.

Although we previously demonstrated that most insertions led to an increase in replication efficiency, the underlying mechanisms remain unclear. Previous investigations relating to nuclear localization sequence (NLS), regulatory sites, sequence-specific patterns inside and outside the PPR (2, 13, 27) have not yielded consistent evidence to explain such phenotype. In addition, despite the incorporation of two human fragments RNA18 or RPL6 in the PPR, no replicative advantage, even slight, was conferred. It is therefore essential to consider host-pathogen interactions at the transcriptional level to better understand the mechanisms of a replicative advantage.

Our global RNA-seq analysis highlighted that VHRVs with enhanced replication induced transcriptional changes, particularly at 168 h post-infection. While PCA showed only partially distinct profiles at 48 h post-infection, VHRVs with and without replicative advantage clustered distinctly at 168 h post-infection.

Functional analysis revealed distinct cellular response kinetics according to VHRVs. At 48 h post-infection, RNF19A, RPS17, ZNF787, and Kernow p6 induce early pathways related to viral replication and innate immunity, whereas these responses are delayed until 168 h for EEF1A1 and KIF1B. Even though RPS17 and Kernow p6 have an inserted fragment derived from the human gene encoding the ribosomal protein S17, the sequences incorporated are not the same, leading to different amino acid (aa) sequences, explaining the differences observed between these two strains. High replication variants activate major immune pathways at 168 h, including interferon signaling and PAMP recognition, reflecting a sustained antiviral response. In contrast, RPL6 and RNA18 did not induce these pathways. Instead, they activated transcriptional or hormonal functions, suggesting either a weak or delayed immune response. A study showed that single-stranded viral RNA alone is sufficient to trigger a robust antiviral interferon response, independent of HEV replication or typical genomic features such as the m7G cap or poly(A) tail (32). This response is IRF3- and IRF7-dependent but independent of RIG-I, MDA5, MAVS, and β-catenin and requires an intact JAK-STAT pathway (32). By analogy, it is possible that RPL6 and RNA18, due to their low replication rate, could activate antiviral pathways through similar unconventional mechanisms**,** potentially contributing to innate immune activation in specific contexts.

The upSet plot analysis showed a larger number of upregulated genes in cells infected with Kernow p6 or RNF19A compared to the other variants. Interestingly, a subset of 25 genes was commonly upregulated across all five VHRVs with a replicative advantage at 168 h post-infection. Seventeen genes correspond to ISGs (OAS1, OAS2, IFIT1, IFIT2). This shared transcriptional signature suggests a robust activation of the antiviral pathways and reflects a common host response to these five VHRV infections. In contrast, downregulated gene profiles were more heterogeneous, with Kernow p6 and RNF19A showing the greatest overlap. It is of note that TPD52L1 was the only gene consistently repressed across all replicative VHRVs which may reflect a conserved mechanism by which viruses modulate host pathways, such as cell proliferation, apoptotic regulation, and calcium signaling (33). TPD52L1 itself is listed among apoptosis-related genes in infectious bronchitis virus in avian models (34). However, to date, T*PD52L1* has not been characterized in the context of HEV infection, and its specific role in antiviral responses remains entirely unexplored. *AREG* commonly expressed for EEF1A1, KIF1B, RPS17, RNF19A, and Kernow p6 has been shown to support persistence mechanisms. In HCV infection, *AREG* expression activates cell survival pathways and is required for efficient viral assembly (35), while, in HBV infection, it enhances the immunosuppressive activity of regulatory T cells (36). Specific analysis of the ISG expression at 168 h post-infection revealed that 25 ISGs were significantly upregulated in cells infected with EEF1A1, RNF19A, ZNF787, KIF1B, and Kernow p6. These findings are consistent with previous studies that reported ISG induction during HEV infection in human hepatocytes and liver tissue (17, 18, 31). RPS17 showed a less pronounced ISG response, with ISG15 as the most induced transcript. Conversely, RNA18 and RPL6 with no replicative advantage failed to induce ISG expression at both early (48 h) and late (168 h) stages of infection, suggesting a weak or absent IFN response. The ISG induction was minimal or absent at 48 h post-infection in infected HepG2/C3A despite already elevated viral RNA levels, suggesting a delayed yet robust IFN-mediated response.

The ISGs act as potent antiviral effectors within innate immunity, targeting multiple stages of the viral life cycle including entry, replication, translation, and egress, through a variety of mechanisms (37). Among the ISGs identified in this study, IFIT1 has been previously shown to restrict HEV replication by inhibiting viral RNA translation (25). However, some ISGs, such as USP18 and SOCS1, inhibit type I interferon signaling, resulting in proviral effects that impair antiviral responses and promote the persistence of viruses such as HCV and HIV (38, 39). Additionally, Viperin can be hijacked by influenza virus and Cytomegalovirus to alter host metabolism and promote viral replication (40), while ADAR1 edits viral RNA from viruses such as dengue virus, aiding immune evasion (41). Lastly, Tetherin restricts viral particle release but is antagonized by Vpu, an accessory protein of HIV-1, which promotes Tetherin degradation and facilitates viral egress, illustrating the complex interplay between ISGs and viruses (42). Lastly, ISG15, a ubiquitin-like protein, has context-dependent roles. It has demonstrated antiviral activity during HIV (43) and HCV infection (44), but it can also enhance viral replication of HCV (45, 46). Sooryanarain and al. (26) showed that the silencing of ISG15 in HEV-transfected cells enhances the antiviral effect of IFN-I, suggesting that ISG15 may act as an immunomodulatory factor that promotes viral replication. This is supported by Wang and al. (47) who reported that ISG15 induction during HEV infection restrains type I IFN responses, potentially facilitating immune evasion and persistence. These observations highlight the complexity of ISG regulation and identify ISG15 as a potential modulator of HEV replication and persistence. Our results similarly show that despite strong ISG induction at 168 h post-infection, VHRVs with a replicative advantage maintain high viral loads. This suggests that ISG upregulation is not only a consequence of enhanced replication but could instead reflect a host immune response that is actively modulated and may be exploited by VHRVs to support their replication.

In this context, it seems important to determine whether VHRVs might trigger a stronger ISG response due to increased replication or conversely that replicative advantage allows them to persist despite a heightened immune response. Such duality has also been observed in chronic HEV infections where persistent viruses can coexist with upregulated ISGs (31). The role of ISGs in controlling or facilitating HEV infection likely depends on specific gene functions and cellular context. Viral genotype could also play a role due to the variability of the sequence between zoonotic HEV3 and HEV4 and human-exclusive HEV1 strains, with amino acids highly conserved in the PPR of HEV3 and HEV4, for instance (48). Whether the sequence of the insertion in the PPR could directly contribute to the strong ISG induction requires further investigations. In the same way, the direct implication of replication on the differential gene expression also requires additional investigations.

Overall, our data provide new insights into the transcriptional landscape associated with HEV VHRVs and support the idea that both viral and host determinants contribute to replication dynamics. The shared transcriptional signatures among high-replicating VHRVs highlight potential targets for further functional validation. Future work should aim to analyze the specific roles of candidate ISGs, such as IFIT1, ISG15, and CXCL10, to better understand their contribution to HEV control or persistence.

In conclusion, this study expands our understanding of HEV host response and identifies host gene candidates that may serve as therapeutic targets for HEV infections.

## MATERIALS AND METHODS

### Cells

The HepG2/C3A (CRL-10741, ATCC) cell line was purchased from the American Type Culture Collection (ATCC). HepG2/C3A cells were incubated at 37°C in an atmosphere containing 5% CO_2_, in Dulbecco’s Modified Eagle Medium (DMEM) supplemented with 10% fetal bovine serum (FBS).

### HEV infection

The transfection of the HepG2/C3A MAVS KO cell line with *in vitro*-transcribed RNA allowed the generation of the viral stock, as described previously (15). A total of 6 × 10^5^ HepG2/C3A cells were distributed in 24-well plates. Cells were infected for 6 h at 35.5°C with 1 × 10^7^ copies of virus and maintained as described previously (15). For cells lysed at 48 h post-infection, the culture medium was not changed. Supernatants at 48 h and 168 h were collected and subsequently clarified by centrifugation (1,000 × *g* for 5 min). A volume of 140 µL of the resulting clear supernatant was used for HEV RNA extraction. For the infection assays, three replicates per experiment were analyzed.

### Real-time RT-qPCR

Supernatant RNA extraction was performed using the QIAamp Viral RNA Mini Kit (Qiagen), according to the manufacturer’s protocol. Quantification of HEV RNA was carried out by real-time quantitative RT-PCR using the Superscript Platinum One-Step Kit (Invitrogen) (49).

### Western blot analysis

HepG2/C3A-infected cells were lysed on ice in a RIPA buffer (Cell Signaling Technology) containing protease inhibitors (Roche). Protein extracts were then separated by electrophoresis on 4%–12% SDS-PAGE gel and transferred onto PVDF membrane (Bio-Rad). To prevent nonspecific binding, the membrane was incubated for 1 h at RT with Odyssey Blocking Buffer (LI-COR Biosciences). Primary antibody incubations were performed overnight at 4°C using monoclonal rabbit anti-IFIT1 (EPR27276-63 clone, AB305301, Abcam) or anti-ISG15 (EPR3446 clone, AB133346, Abcam), or a monoclonal mouse antibody against GAPDH (sc-47724, Santa Cruz Biotechnology), diluted in the blocking buffer. After three washes with TBS-T, the membranes were incubated for 1 h at RT with IRDye-labeled secondary antibodies: goat anti-mouse IgG secondary antibody IRDye 680RD (LI-COR, Biosciences) or donkey anti-rabbit IgG secondary antibody IRDye 800CW (LI-COR, Biosciences). Membranes were revealed using the Odyssey Fc Imaging System, and image analysis was performed using Image Studio Lite version 5.0. The ImageJ software (version 1.54c) was used to quantify bands intensities (ImageJ is authored by Wayne Rasband, Research Services Branch, National Institute of Mental Health, Bethesda, Maryland, USA). Briefly, images were formatted in 16-bit images and analyzed with the “Gel” function of ImageJ. The signal obtained for the protein of interest was reported to that obtained for GAPDH. Each ratio was reported to the value obtained for the Kernow p6 sample.

### Measurement of CXCL10 protein levels

IP-10 (CXCL10) concentrations in cell culture supernatants were quantified using the V-PLEX Plus Chemokine Panel 1 Human (Gen 8) Kit (K15705G, Meso Scale Discovery, Rockville, MD, USA), according to the manufacturer’s instructions. The assay sensitivity ranged from 0.12 to 1.240 pg/mL.

### RNA sequencing

#### RNA extraction

Infected HepG2/C3A were washed with PBS, trypsinized, and pelleted by centrifugation at 300 × *g* for 5 min. Cell pellets were resuspended in 500 µL of the lysis buffer and then passed through a Qiashredder column to homogenize the cell lysates. Columns were centrifuged at maximum speed for 2 min. Total RNA was extracted from eluted cell lysates using the RNeasy Mini Kit (Qiagen) according to the manufacturer’s instructions. The RNA was eluted twice in 30 µL RNase-free water to maximize yield. Purified RNA samples were stored at −80°C until further analysis.

### Library construction

#### RNA quantification and quality control

Total RNA was quantified using the Qubit RNA HS Assay Kit and the Qubit Fluorometer (Thermo Fisher Scientific). RNA integrity and quality were assessed using the Agilent 4150 TapeStation system with the High Sensitivity RNA ScreenTape Kit (Agilent Technologies). RNA samples were normalized to a final amount of 1000 ng prior to library preparation.

### Library preparation

Stranded total RNA libraries were prepared using the TruSeq Stranded Total RNA Library Prep Gold Kit (Illumina), according to the manufacturer’s protocol with minor modifications. Ribosomal RNA (rRNA) and mitochondrial rRNA were depleted using Ribo-Zero Gold beads (Illumina). The remaining RNA was enzymatically fragmented and reverse-transcribed to generate cDNA while preserving strand orientation. After adapter ligation, libraries were amplified by PCR. All purification steps using AMPure XP beads (Beckman Coulter) were modified by reducing the bead drying time from 15 min to 2–6 min, assessed visually. Following the final amplification step, libraries were subjected to an additional purification and size selection step to improve quality.

### Library quantification and quality control

Final libraries were quantified using the Qubit 1× dsDNA HS Assay Kit on the Qubit Fluorometer (Thermo Fisher Scientific), and their size distribution and quality were evaluated using the Agilent 4150 TapeStation with the High Sensitivity D5000 ScreenTape Kit (Agilent Technologies).

### Sequencing

Libraries were equimolarly pooled, and RNA sequencing was then performed on one S4 lane of the Illumina NovaSeq 6000 instrument (Illumina, San Diego, USA), at the GeT-PlaGe site of the Genome and Transcriptome core facility (Toulouse, France), using the NovaSeq 6000 S4 v1.5 Reagent Kit (300 cycles) and a paired-end 2 × 150 pb strategy. This resulted in 38 to 59 million pairs of raw reads per library.

### Bioinformatic analysis

#### Quantification of RNA expression

Raw sequencing reads were processed using the nf-core RNAseq pipeline v.3.12 developed using Nextflow (50, 51). Briefly, this pipeline trims adapters and removes low-quality sequences using Cutadapt v.3.4 wrapped with Trimgalore v.0.6.7 (52, 53). rRNAs are filtered out using SortMeRNA v.4.3.4 (54). STAR v.2.7.9a (55) then aligns trimmed reads to a chimeric assembly composed of the *Homo sapiens* GRCh38 assembly and the complete genome of Hepatitis E virus clone pSK-HEV-2 (GenBank: AF444002.1) (56). Gene annotation includes *Homo sapiens* annotation from Ensembl (57) release 110 and Hepatitis E virus ORFs. Parameters used to run STAR were:

--outSAMstrandField intronMotif --quantTranscriptomeBan Singleend --runRNGseed 0 --twopassMode Basic --quantMode TranscriptomeSAM.

Finally, Salmon v.1.5.2 (58) quantifies reads with parameter --libType=ISR.

#### Differential expression analyses

Raw count matrix was imported into R using the R package DESeq2 v.1.42.0 (59). Design parameter from the DESeqDataSetFromTximport() function was set to ~condition, where condition indicates the strain used and the time since infection. Expressed genes were identified as genes with at least 10 raw counts for two samples of the same condition. We used DESeq() and result() functions, with default parameters, to normalize data and run DEA, respectively.

PCA was performed using PCA() function on the counts after variance stabilization transformation performed with the vst() function. Heatmaps were generated with the pheatmap() function of the pheatmap package and scale = “row” as parameter. Upset plots were created with the upset() function of the UpsetR R package.

#### Enrichment analyses

We ran the enrichment analyses with the R package clusterProfiler v.4.10.0 (60) using gene sets from the Gene Ontology (GO.db_3.18.0) database. Over-representation analyses were performed on differentially expressed genes (DEG) defined as genes with an adjusted *P* value below 0.05 and an absolute log2 fold change greater than 1. The background was set as all expressed genes and was specified using the universe parameter of enrichGO function.

Gene set enrichment analyses were performed on a gene list ranked by log2 fold change extracted from DESeq2 object. The enrichment was tested on the ISG gene list extracted from reference 61.

## Data Availability

RNA-seq data have been deposited at ArrayExpress (https://www.ebi.ac.uk/biostudies/arrayexpress/studies). The accession number for the raw and processed data for RNAseq generated in this study is E-MTAB-16873. Any additional information required to reanalyze the data reported in this paper can be obtained from the corresponding author upon request.

## SUPPLEMENTAL MATERIAL

Supplemental material spectrum.00600-26-s0001.docx Fig. S1 to S6 and Tables S1 to S16.

ASM does not own the copyrights to Supplemental Material that may be linked to, or accessed through, an article. The authors have granted ASM a non-exclusive, world-wide license to publish the Supplemental Material files. Please contact the corresponding author directly for reuse.
