# Supplementary material for: Host-interferon-stimulated gene response to virus-host recombinant variants of hepatitis E virus and enhanced viral replication
Source: Microbiol Spectr. 2026 Jul 13;14(8):e00600-26. doi: 10.1128/spectrum.00600-26 (PMC13435683; doi:10.1128/spectrum.00600-26)
Supplement: Supplementary file 1 [file spectrum.00600-26-s0001.docx]

**Host-Interferon-Stimulated Gene Response to Virus-Host Recombinant Variants of Hepatitis E Virus and Enhanced Viral Replication**

**Authors**

Olivia Paronetto^1^, Claire Allioux^1^, Margot Zahm^1^, Anne Alicia Gonzalez^1^, Nicolas Jeanne^1,2^, Adeline Chaubet^1^, Nived Collercandy^1,3^, Florence Abravanel^1,2^, Émeline Lhuillier^4,5^, Sabine Chapuy-Regaud^1,2^, Jacques Izopet^1,2^ and Sébastien Lhomme^1,2^

**Affiliations**

^1^Institut Toulousain des Maladies Infectieuses et Inflammatoires (Infinity), UMR 5051 (CNRS), UMR 1291 (INSERM), Université́ de Toulouse, Toulouse, France

^2^Laboratoire de Virologie, Hôpital Purpan, CHU Toulouse, Toulouse, France

^3^Service des maladies infectieuses et tropicales, Hôpital Purpan, CHU Toulouse, Toulouse, France

^4^I2MC, INSERM UMR 1297, Toulouse, France

^5^GeT-Santé, Plateforme Génome et Transcriptome, GenoToul, Toulouse, France.

**Abstract word count:** 235**/250**

**Text word count:** 4951**/5000**

**Importance word count:** 126**/150**

**Keywords:** Hepatitis E virus, virus-host recombinant variants, RNA sequencing, interferon stimulated gene, transcriptomic.

**Supplementary Information**

A

B

**Figure S1: Gene Ontology enrichment analysis of differentially expressed genes in HepG2/C3A cells infected with RPS17.** Infection of cells with RPS17 at 48 h (A) and 168 h (B) post-infection. Analyses were performed using clusterProfiler (v.4.10.0) based on the GO Biological Process database.

A

B

**Figure S2: Gene Ontology enrichment analysis of differentially expressed genes in HepG2/C3A cells infected with ZNF787.** Infection of cells with ZNF787 at 48 h (A) and 168 h (B) post-infection. Analyses were performed using clusterProfiler (v.4.10.0) based on the GO Biological Process database.

A

B

**Figure S3: Gene Ontology enrichment analysis of differentially expressed genes in HepG2/C3A cells infected with Kernow p6.** Infection of cells with Kernow p6 at 48 h (A) and 168 h (B) post-infection. Analyses were performed using clusterProfiler (v.4.10.0) based on the GO Biological Process database.

A

B

**Figure S4: Gene Ontology enrichment analysis of differentially expressed genes in HepG2/C3A cells infected with EEF1A1.** Infection of cells with EEF1A1 at 48 h (A) and 168 h (B) post-infection. Analyses were performed using clusterProfiler (v.4.10.0) based on the GO Biological Process database.

**Figure S5: Gene Ontology enrichment analysis of differentially expressed genes in HepG2/C3A cells infected with KIF1B.** Infection of cells with KIF1B at 168 h post-infection. Analyses were performed using clusterProfiler (v.4.10.0) based on the GO Biological Process database.

**Figure S6: Gene Ontology enrichment analysis of differentially expressed genes in HepG2/C3A cells infected with RNA18.** Infection of cells with RNA18 at 168 h post-infection. Analyses were performed using clusterProfiler (v.4.10.0) based on the GO Biological Process database.

**Table S1: List of the 89 upregulated genes identified for Kernow p6 at 168h post-infection.** For each gene, the table provides the Ensembl gene ID, the gene symbol and the complete name.

| GENE ID |  | GENE SYMBOL | GENE NAME |  |  |  | |  | |  |
| --- | --- | --- | --- | --- | --- | --- | --- | --- | --- | --- |
| ENSG00000075275 | | CELSR1 | Cadherin EGF LAG Seven-Pass G-Type Receptor 1 | | | | | |  | |
| ENSG00000087074 | | PPP1R15A | Protein Phosphatase 1 Regulatory Subunit 15A | | | | | |  | |
| ENSG00000100036 | | SLC35E4 | Solute Carrier Family 35 Member E4 | | | |  | |  | |
| ENSG00000100055 | | CYTH4 | Cytohesin 4 |  |  |  | |  | |  |
| ENSG00000100906 | | NFKBIA | Nuclear Factor Kappa B Inhibitor Alpha | | | |  | |  | |
| ENSG00000101443 | | WFDC2 | WAP Four-Disulfide Core Domain 2 | | | |  | |  | |
| ENSG00000101574 | | METTL4 | Methyltransferase Like 4 | |  |  | |  | |  |
| ENSG00000104490 | | NCALD | Neurocalcin Delta | |  |  | |  | |  |
| ENSG00000104856 | | RELB | RELB Proto-Oncogene, NF-KB Subunit | | | |  | |  | |
| ENSG00000105523 | | FAM83E | Family With Sequence Similarity 83 Member E | | | | | |  | |
| ENSG00000106631 | | MYL7 | Myosin Light Chain 7 | |  |  | |  | |  |
| ENSG00000107186 | | MPDZ | Multiple PDZ Domain Crumbs Cell Polarity Complex Component | | | | | | | |
| ENSG00000116667 | | C1orf21 | Chromosome 1 Open Reading Frame 21 | | | |  | |  | |
| ENSG00000119508 | | NR4A3 | Nuclear Receptor Subfamily 4 Group A Member 3 | | | | | |  | |
| ENSG00000120129 | | DUSP1 | Dual Specificity Phosphatase 1 | | | |  | |  | |
| ENSG00000128573 | | FOXP2 | Forkhead Box P2 | |  |  | |  | |  |
| ENSG00000129116 | | PALLD | Palladin, Cytoskeletal Associated Protein | | | |  | |  | |
| ENSG00000130783 | | CCDC62 | Coiled-Coil Domain Containing 62 | | | |  | |  | |
| ENSG00000132357 | | CARD6 | Caspase Recruitment Domain Family Member 6 | | | | | |  | |
| ENSG00000134343 | | ANO3 | Anoctamin3 |  |  |  | |  | |  |
| ENSG00000134548 | | SPX | Spexin |  |  |  | |  | |  |
| ENSG00000134871 | | COL4A2 | Collagen Type IV Alpha 2 Chain | | | |  | |  | |
| ENSG00000135625 | | EGR4 | Early Growth Response 4 | |  |  | |  | |  |
| ENSG00000137094 | | DNAJB5 | DnaJ Heat Shock Protein Family (Hsp40) Member B5 | | | | | |  | |
| ENSG00000137819 | | PAQR5 | Progestin And AdipoQ Receptor Family Member 5 | | | | | |  | |
| ENSG00000138166 | | DUSP5 | Dual Specificity Phosphatase 5 | | | |  | |  | |
| ENSG00000138172 | | CALHM2 | Calcium Homeostasis Modulator Family Member 2 | | | | | |  | |
| ENSG00000143507 | | DUSP10 | Dual Specificity Phosphatase 10 | | | |  | |  | |
| ENSG00000144893 | | MED12L | Mediator Complex Subunit 12 Like | | | |  | |  | |
| ENSG00000145198 | | VWA5B2 | Von Willebrand Factor A Domain Containing 5B2 | | | | | |  | |
| ENSG00000146556 | | WASH2P | WASH Complex Subunit 2, Pseudogene | | | |  | |  | |
| ENSG00000147168 | | IL2RG | Interleukin 2 Receptor Subunit Gamma | | | |  | |  | |
| ENSG00000149201 | | CCDC81 | Coiled-Coil Domain Containing 81 | | | |  | |  | |
| ENSG00000149599 | | DUSP15 | Dual Specificity Phosphatase 15 | | | |  | |  | |
| ENSG00000150873 | | C2orf50 | Chromosome 2 Open Reading Frame 50 | | | |  | |  | |
| ENSG00000153443 | | UBALD1 | UBA Like Domain Containing 1 | | | |  | |  | |
| ENSG00000153933 | | DGKE | Diacylglycerol Kinase Epsilon | | | |  | |  | |
| ENSG00000154153 | | RETREG1 | Reticulophagy Regulator 1 | |  |  | |  | |  |
| ENSG00000156127 | | BATF | Basic Leucine Zipper ATF-Like Transcription Factor | | | | | |  | |
| ENSG00000159713 | | TPPP3 | Tubulin Polymerization Promoting Protein Family Member 3 | | | | | | | |
| ENSG00000160072 | | ATAD3B | ATPase Family AAA Domain Containing 3B | | | |  | |  | |
| ENSG00000160190 | | SLC37A1 | Solute Carrier Family 37 Member 1 | | | |  | |  | |
| ENSG00000160200 | | CBS | Cystathionine Beta-Synthase | | | |  | |  | |
| ENSG00000161180 | | CCDC116 | Coiled-Coil Domain Containing 116 | | | |  | |  | |
| ENSG00000163131 | | CTSS | Cathepsin S |  |  |  | |  | |  |
| ENSG00000163220 | | S100A9 | S100 Calcium Binding Protein A9 | | | |  | |  | |
| ENSG00000166741 | | NNMT | Nicotinamide N-Methyltransferase | | | |  | |  | |
| ENSG00000169548 | | ZNF280A | Zinc Finger Protein 280A | |  |  | |  | |  |
| ENSG00000172216 | | CEBPB | CCAAT Enhancer Binding Protein Beta | | | |  | |  | |
| ENSG00000174276 | | ZNHIT2 | Zinc Finger HIT-Type Containing 2 | | | |  | |  | |
| ENSG00000175175 | | PPM1E | Protein Phosphatase, Mg2+/Mn2+ Dependent 1E | | | | | |  | |
| ENSG00000177606 | | JUN | Jun Proto-Oncogene, AP-1 Transcription Factor Subunit | | | | | |  | |
| ENSG00000178033 | | CALHM5 | Calcium Homeostasis Modulator Family Member 5 | | | | | |  | |
| ENSG00000178776 | | C5orf46 | Chromosome 5 Open Reading Frame 46 | | | |  | |  | |
| ENSG00000183458 | | PKD1P3 | Polycystic Kidney Disease 1 Pseudogene 3 | | | | | |  | |
| ENSG00000184557 | | SOCS3 | Suppressor Of Cytokine Signaling 3 | | | |  | |  | |
| ENSG00000188290 | | HES4 | Hes Family BHLH Transcription Factor 4 | | | |  | |  | |
| ENSG00000189325 | | BNIP5 | BCL2 Interacting Protein 5 | |  |  | |  | |  |
| ENSG00000197943 | | PLCG2 | Phospholipase C Gamma 2 | |  |  | |  | |  |
| ENSG00000206341 | | HLA-H | Major Histocompatibility Complex, Class I, H (Pseudogene) | | | | | | | |
| ENSG00000206503 | | HLA-A | Major Histocompatibility Complex, Class I, A | | | | | |  | |
| ENSG00000214212 | | C19orf38 | Chromosome 19 Open Reading Frame 38 | | | |  | |  | |
| ENSG00000215182 | | MUC5AC | Mucin 5AC, Oligomeric Mucus/Gel-Forming | | | | | |  | |
| ENSG00000220412 | | PTPN11P3 | Protein Tyrosine Phosphatase Non-Receptor Type 11 Pseudogene 3 | | | | | | | |
| ENSG00000225345 | | SNX18P3 | Sorting Nexin 18 Pseudogene 3 | | | |  | |  | |
| ENSG00000234832 | | NXT1-AS1 | NXT1 Antisense RNA 1 | |  |  | |  | |  |
| ENSG00000234840 | | LINC01239 | Long Intergenic Non-Protein Coding RNA 1239 | | | | | |  | |
| ENSG00000237181 | | PRKAR1B-AS1 | PRKAR1B Antisense RNA 1 | |  |  | |  | |  |
| ENSG00000246985 | | SOCS2-AS1 | SOCS2 Antisense RNA 1 | | | | | |  | |
| ENSG00000247324 | | ENSG00000247324 | [Non-characterized/Unannotated ENSG ID] | | | | | |  | |
| ENSG00000250251 | | PKD1P6 | Polycystic Kidney Disease 1 Pseudogene 6 | | | |  | |  | |
| ENSG00000255398 | | HCAR3 | Hydroxycarboxylic Acid Receptor 3 | |  |  | |  | |  |
| ENSG00000255874 | | PRECSIT | [Limited annotation] | | | |  | |  | |
| ENSG00000256061 | | DNAAF4 | Dynein Axonemal Assembly Factor 4 | |  |  | |  | |  |
| ENSG00000259856 | | RAB43P1 | RAB43 Pseudogene 1 | | | | | |  | |
| ENSG00000263766 | | KPNB1-DT | KPNB1 Divergent Transcript (likely lncRNA) | | | | | |  | |
| ENSG00000264666 | | ENSG00000264666 | [Non-characterized/Unannotated ENSG ID] | | | | | |  | |
| ENSG00000269897 | | COMMD3-BMI1 | Read-through transcript between COMMD3 and BMI1 | | | | | |  | |
| ENSG00000270580 | | PKD1P6-NPIPP1 | Read-through transcript between PKD1P6 and NPIPP1 | | | |  | |  | |
| ENSG00000271943 | | TPM4P2 | Tropomyosin 4 Pseudogene 2 | | | | | |  | |
| ENSG00000273151 | | ENSG00000273151 | [Non-characterized/Unannotated ENSG ID] | | | | | |  | |
| ENSG00000275202 | | ENSG00000275202 | [Non-characterized/Unannotated ENSG ID] | | | | | |  | |
| ENSG00000285565 | | ENSG00000285565 | [Non-characterized/Unannotated ENSG ID] | | | |  | |  | |
| ENSG00000285723 | | MKKS | McKusick-Kaufman Syndrome (BBS6) | | | | | |  | |
| ENSG00000286231 | | ENSG00000286231 | [Non-characterized/Unannotated ENSG ID] | | | | | |  | |
| ENSG00000287265 | | ENSG00000287265 | [Non-characterized/Unannotated ENSG ID] | | | |  | |  | |
| ENSG00000290862 | | NMRAL2P | NmrA Like Redox Sensor 2 Pseudogene | | | | | |  | |
| ENSG00000291067 | | ENSG00000291067 | [Non-characterized/Unannotated ENSG ID] | | | | | |  | |
| ENSG00000291273 | | ENSG00000291273 | [Non-characterized/Unannotated ENSG ID] | | | | | |  | |

**Table S2: List of the 43 upregulated genes identified for RNF19A at 168h post-infection.** For each gene, the table provides the Ensembl gene ID, the gene symbol and the complete name.

| GENE ID |  | GENE SYMBOL | GENE NAME |  |  |  |
| --- | --- | --- | --- | --- | --- | --- |
| ENSG00000011332 | | DPF1 | Double PHD Fingers 1 | |  |  |
| ENSG00000034677 | | RNF19A | Ring Finger Protein 19A, E3 Ubiquitin Protein Ligase | | | |
| ENSG00000088881 | | EBF4 | Early B-Cell Factor 4 | |  |  |
| ENSG00000101670 | | LIPG | Lipase G, Endothelial Type | |  |  |
| ENSG00000105697 | | HAMP | Hepcidin Antimicrobial Peptide | | |  |
| ENSG00000112486 | | CCR6 | C-C Motif Chemokine Receptor 6 | | |  |
| ENSG00000114631 | | PODXL2 | Podocalyxin Like 2 | |  |  |
| ENSG00000116183 | | PAPPA2 | Pappalysin 2 |  |  |  |
| ENSG00000116254 | | CHD5 | Chromodomain Helicase DNA Binding Protein 5 | | | |
| ENSG00000121900 | | TMEM54 | Transmembrane Protein 54 | |  |  |
| ENSG00000128604 | | IRF5 | Interferon Regulatory Factor 5 | | |  |
| ENSG00000128872 | | TMOD2 | Tropomodulin 2 | |  |  |
| ENSG00000131370 | | SH3BP5 | SH3 Domain Binding Protein 5 | | |  |
| ENSG00000146530 | | VWDE | Von Willebrand Factor D and EGF Domains | | | |
| ENSG00000151882 | | CCL28 | C-C Motif Chemokine Ligand 28 | | |  |
| ENSG00000157578 | | LCA5L | Lebercilin 5 Like | |  |  |
| ENSG00000159753 | | CARMIL2 | Capping Actin Protein Regulator and Myosin 1 Linker 2 | | | |
| ENSG00000160606 | | TLCD1 | TLC Domain Containing 1 | |  |  |
| ENSG00000165521 | | EML5 | Echinoderm Microtubule Associated Protein Like 5 | | | |
| ENSG00000169989 | | TIGD4 | Tigger Transposable Element Derived 4 | | |  |
| ENSG00000170581 | | STAT2 | Signal Transducer and Activator of Transcription 2 | | | |
| ENSG00000175643 | | RMI2 | RecQ Mediated Genome Instability 2 | | |  |
| ENSG00000177570 | | SAMD12 | Sterile Alpha Motif Domain Containing 12 | | |  |
| ENSG00000186648 | | CARMIL3 | Capping Actin Protein Regulator and Myosin 1 Linker 3 | | | |
| ENSG00000187699 | | C2orf88 | Chromosome 2 Open Reading Frame 88 | | |  |
| ENSG00000189233 | | NUGGC | Nuclear GTPase, G Protein Connecting | | |  |
| ENSG00000196776 | | CD47 | CD47 Molecule | |  |  |
| ENSG00000198754 | | OXCT2 | 3-Oxoacid CoA-Transferase 2 | | |  |
| ENSG00000205181 | | LINC00654 | Long Intergenic Non-Protein Coding RNA 654 | | | |
| ENSG00000227192 | | ENSG00000227192 | Uncharacterized / Non-coding transcript | | |  |
| ENSG00000236830 | | CBR3-AS1 | CBR3 Antisense RNA 1 | |  |  |
| ENSG00000257017 | | HP | Haptoglobin |  |  |  |
| ENSG00000270022 | | ENSG00000270022 | Uncharacterized / Non-coding transcript | | |  |
| ENSG00000272505 | | ENSG00000272505 | Uncharacterized / Non-coding transcript | | |  |
| ENSG00000272636 | | DOC2B | Double C2 Domain Beta | |  |  |
| ENSG00000277161 | | PIGW | Phosphatidylinositol Glycan Anchor Biosynthesis Class W | | | |
| ENSG00000284194 | | SCO2 | SCO2 Cytochrome C Oxidase Assembly Factor | | | |
| ENSG00000287979 | | ENSG00000287979 | Uncharacterized / Non-coding transcript | | |  |
| ENSG00000288591 | | ENSG00000288591 | Uncharacterized / Non-coding transcript | | |  |
| ENSG00000289554 | | ENSG00000289554 | Uncharacterized / Non-coding transcript | | |  |
| ENSG00000289754 | | ENSG00000289754 | Uncharacterized / Non-coding transcript | | |  |
| ENSG00000290386 | | ENSG00000290386 | Uncharacterized / Non-coding transcript | | |  |
| ENSG00000291128 | | APOC1P1 | Apolipoprotein C1 Pseudogene 1 | | |  |

**Table S3: List of the 19 upregulated genes identified for EEF1A1 at 168h post-infection.** For each gene, the table provides the Ensembl gene ID, the gene symbol and the complete name.

| GENE ID |  | GENE SYMBOL | GENE NAME |  |  |  |  |
| --- | --- | --- | --- | --- | --- | --- | --- |
| ENSG00000049323 | | LTBP1 | Latent Transforming Growth Factor Beta Binding Protein 1 | | | | |
| ENSG00000064300 | | NGFR | Nerve Growth Factor Receptor | | |  |  |
| ENSG00000065413 | | ANKRD44 | Ankyrin Repeat Domain 44 | |  |  |  |
| ENSG00000129028 | | THAP10 | THAP Domain Containing 10 | | |  |  |
| ENSG00000134013 | | LOXL2 | Lysyl Oxidase Like 2 | |  |  |  |
| ENSG00000135074 | | ADAM19 | ADAM Metallopeptidase Domain 19 | | |  |  |
| ENSG00000138119 | | MYOF | Myoferlin |  |  |  |  |
| ENSG00000143061 | | IGSF3 | Immunoglobulin Superfamily Member 3 | | |  |  |
| ENSG00000148677 | | ANKRD1 | Ankyrin Repeat Domain 1 | |  |  |  |
| ENSG00000158023 | | CFAP251 | Cilia and Flagella Associated Protein 251 | | |  |  |
| ENSG00000159917 | | ZNF235 | Zinc Finger Protein 235 | |  |  |  |
| ENSG00000164761 | | TNFRSF11B | TNF Receptor Superfamily Member 11B | | |  |  |
| ENSG00000167767 | | KRT80 | Keratin 80 |  |  |  |  |
| ENSG00000174099 | | MSRB3 | Methionine Sulfoxide Reductase B3 | | |  |  |
| ENSG00000176809 | | LRRC37A3 | Leucine Rich Repeat Containing 37A3 | | |  |  |
| ENSG00000178386 | | ZNF223 | Zinc Finger Protein 223 | |  |  |  |
| ENSG00000204540 | | PSORS1C1 | Psoriasis Susceptibility 1 Candidate 1 | | |  |  |
| ENSG00000215483 | | LINC00598 | Long Intergenic Non-Protein Coding RNA 598 | | | |  |
| ENSG00000255774 | | LINC02747 | Long Intergenic Non-Protein Coding RNA 2747 | | | |  |

**Table S4: List of the 11 upregulated genes identified for ZNF787 at 168h post-infection.** For each gene, the table provides the Ensembl gene ID, the gene symbol and the complete name.

| GENE ID |  | GENE SYMBOL | | GENE NAME |  |  |  |
| --- | --- | --- | --- | --- | --- | --- | --- |
| ENSG00000157087 | | ATP2B2 |  | Plasma Membrane Calcium-Transporting ATPase 2 | | | |
| ENSG00000173867 | | ENSG00000173867 | | Uncharacterized / Non-coding transcript | | |  |
| ENSG00000184226 | | PCDH9 |  | Protocadherin 9 | |  |  |
| ENSG00000186472 | | PCLO |  | Piccolo Presynaptic Cytomatrix Protein | | |  |
| ENSG00000187122 | | SLIT1 |  | Slit Guidance Ligand 1 | |  |  |
| ENSG00000244198 | | ARHGEF35-AS1 | | ARHGEF35 Antisense RNA 1 (lncRNA) | | |  |
| ENSG00000257335 | | MGAM |  | Maltase-Glucoamylase | |  |  |
| ENSG00000285330 | | ENSG00000285330 | | Uncharacterized / Non-coding transcript | | |  |
| ENSG00000288550 | | ENSG00000288550 | | Uncharacterized / Non-coding transcript | | |  |
| ENSG00000289027 | | ENSG00000289027 | | Uncharacterized / Non-coding transcript | | |  |
| ENSG00000290041 | | ENSG00000290041 | | Uncharacterized / Non-coding transcript | | |  |

**Table S5: List of the 155 upregulated genes identified for RNF19A and Kernow p6 at 168h post-infection.** This table includes genes that are commonly significantly upregulated for RNF19A and Kernow p6. For each gene, the table provides the Ensembl gene ID, the gene symbol and the complete name.

| GENE ID |  | GENE SYMBOL | GENE NAME |  |  |  |  |  |  |
| --- | --- | --- | --- | --- | --- | --- | --- | --- | --- |
| ENSG00000002549 | | LAP3 | Leucine aminopeptidase 3 | |  |  |  |  |  |
| ENSG00000006062 | | MAP3K14 | [Mitogen-activated protein kinase kinase kinase 14](https://www.ncbi.nlm.nih.gov/gene?Cmd=DetailsSearch&Db=gene&Term=9020&utm_source=chatgpt.com) | | | |  |  |  |
| ENSG00000010810 | | FYN | FYN proto-oncogene, Src family tyrosine kinase | | | |  |  |  |
| ENSG00000011422 | | PLAUR | Plasminogen activator, urokinase receptor | | | |  |  |  |
| ENSG00000049249 | | TNFRSF9 | TNF receptor superfamily member 9 | | |  |  |  |  |
| ENSG00000060982 | | BCAT1 | Branched chain amino acid transaminase 1 | | | |  |  |  |
| ENSG00000065320 | | NTN1 | Netrin 1 |  |  |  |  |  |  |
| ENSG00000070669 | | ASNS | Asparagine synthetase | |  |  |  |  |  |
| ENSG00000077522 | | ACTN2 | Actinin alpha 2 | |  |  |  |  |  |
| ENSG00000078114 | | NEBL | Nebulette |  |  |  |  |  |  |
| ENSG00000085741 | | WNT11 | Wnt family member 11 | |  |  |  |  |  |
| ENSG00000095752 | | IL11 | Interleukin 11 | |  |  |  |  |  |
| ENSG00000100342 | | APOL1 | Apolipoprotein L1 | |  |  |  |  |  |
| ENSG00000100867 | | DHRS2 | Dehydrogenase/reductase member 2 | | |  |  |  |  |
| ENSG00000100911 | | PSME2 | Proteasome activator subunit 2 | | |  |  |  |  |
| ENSG00000101255 | | TRIB3 | Tribbles pseudokinase 3 | |  |  |  |  |  |
| ENSG00000101347 | | SAMHD1 | SAM and HD domain containing deoxynucleoside triphosphate triphosphohydrolase 1 | | | | | | |
| ENSG00000103966 | | EHD4 | EH domain containing 4 | |  |  |  |  |  |
| ENSG00000105499 | | PLA2G4C | Phospholipase A2 group 4C | |  |  |  |  |  |
| ENSG00000106785 | | TRIM14 | Tripartite motif containing 14 | | |  |  |  |  |
| ENSG00000107731 | | UNC5B | Unc-5 netrin receptor B | |  |  |  |  |  |
| ENSG00000108679 | | LGALS3BP | Galectin 3 binding protein | |  |  |  |  |  |
| ENSG00000109452 | | INPP4B | Inositol polyphosphate-4-phosphatase type II B | | | |  |  |  |
| ENSG00000109586 | | GALNT7 | Polypeptide N-acetylgalactosaminyltransferase 7 | | | |  |  |  |
| ENSG00000111837 | | MAK | Male germ cell-associated kinase | | |  |  |  |  |
| ENSG00000112343 | | TRIM38 | Tripartite motif containing 38 | | |  |  |  |  |
| ENSG00000112576 | | CCND3 | Cyclin D3 |  |  |  |  |  |  |
| ENSG00000114450 | | GNB4 | G protein subunit β 4 | |  |  |  |  |  |
| ENSG00000116663 | | FBXO6 | F-box protein 6 | |  |  |  |  |  |
| ENSG00000117643 | | MAN1C1 | Mannosidase alpha class 1 C member 1 | | |  |  |  |  |
| ENSG00000120738 | | EGR1 | Early growth response 1 | |  |  |  |  |  |
| ENSG00000122694 | | GLIPR2 | GLI pathogenesis-related 2 | |  |  |  |  |  |
| ENSG00000123689 | | G0S2 | G0/G1 switch gene 2 | |  |  |  |  |  |
| ENSG00000124882 | | EREG | Epiregulin |  |  |  |  |  |  |
| ENSG00000125347 | | IRF1 | Interferon regulatory factor 1 | | |  |  |  |  |
| ENSG00000125740 | | FOSB | FBJ murine osteosarcoma viral oncogene homolog B | | | |  |  |  |
| ENSG00000127666 | | TICAM1 | Toll-like receptor adaptor molecule 1 | | |  |  |  |  |
| ENSG00000128165 | | ADM2 | Adrenomedullin 2 | |  |  |  |  |  |
| ENSG00000128284 | | APOL3 | Apolipoprotein L3 | |  |  |  |  |  |
| ENSG00000128285 | | MCHR1 | Melanin-concentrating hormone receptor 1 | | | |  |  |  |
| ENSG00000128335 | | APOL2 | Apolipoprotein L2 | |  |  |  |  |  |
| ENSG00000128342 | | LIF | Leukemia inhibitory factor | |  |  |  |  |  |
| ENSG00000128965 | | CHAC1 | ChaC glutathione-specific gamma-glutamylcyclotransferase 1 | | | | |  |  |
| ENSG00000131979 | | GCH1 | GTP cyclohydrolase 1 | |  |  |  |  |  |
| ENSG00000135604 | | STX11 | Syntaxin 11 |  |  |  |  |  |  |
| ENSG00000136826 | | KLF4 | Kruppel like factor 4 | |  |  |  |  |  |
| ENSG00000137673 | | MMP7 | Matrix metallopeptidase 7 | |  |  |  |  |  |
| ENSG00000139269 | | INHBE | Inhibin subunit beta E | |  |  |  |  |  |
| ENSG00000139278 | | GLIPR1 | GLI pathogenesis-related 1 | |  |  |  |  |  |
| ENSG00000140022 | | STON2 | Stonin 2 |  |  |  |  |  |  |
| ENSG00000140044 | | JDP2 | Jun dimerization protein 2 | |  |  |  |  |  |
| ENSG00000140105 | | WARS1 | Tryptophanyl-tRNA synthetase 1 | | |  |  |  |  |
| ENSG00000140379 | | BCL2A1 | BCL2 related protein A1 | |  |  |  |  |  |
| ENSG00000140464 | | PML | Promyelocytic leukemia | |  |  |  |  |  |
| ENSG00000140465 | | CYP1A1 | Cytochrome P450 family 1 subfamily A member 1 | | | |  |  |  |
| ENSG00000141971 | | MVB12A | Multivesicular body subunit 12A | | |  |  |  |  |
| ENSG00000144488 | | ESPNL | Espin-like |  |  |  |  |  |  |
| ENSG00000144596 | | GRIP2 | Glutamate receptor interacting protein 2 | | |  |  |  |  |
| ENSG00000144837 | | PLA1A | Phospholipase A1 member A | | |  |  |  |  |
| ENSG00000144843 | | ADPRH | ADP-ribosylarginine hydrolase | | |  |  |  |  |
| ENSG00000151012 | | SLC7A11 | Solute carrier family 7 member 11 | | |  |  |  |  |
| ENSG00000152137 | | HSPB8 | Heat shock protein family B (small) member 8 | | | |  |  |  |
| ENSG00000159403 | | C1R | Complement C1r | |  |  |  |  |  |
| ENSG00000160710 | | ADAR | Adenosine deaminase RNA specific | | |  |  |  |  |
| ENSG00000160888 | | IER2 | Immediate early response 2 | | |  |  |  |  |
| ENSG00000161791 | | FMNL3 | Formin like 3 |  |  |  |  |  |  |
| ENSG00000162998 | | FRZB | Frizzled-related protein | |  |  |  |  |  |
| ENSG00000163393 | | SLC22A15 | Solute carrier family 22 member 15 | | |  |  |  |  |
| ENSG00000163545 | | NUAK2 | NUAK family kinase 2 | |  |  |  |  |  |
| ENSG00000163577 | | EIF5A2 | Eukaryotic translation initiation factor 5A2 | | | |  |  |  |
| ENSG00000163644 | | PPM1K | Protein phosphatase, Mg2+/Mn2+ dependent 1K | | | |  |  |  |
| ENSG00000163684 | | RPP14 | Ribonuclease P/MRP subunit p14 | | |  |  |  |  |
| ENSG00000163734 | | CXCL3 | C-X-C motif chemokine ligand 3 | | |  |  |  |  |
| ENSG00000164136 | | IL15 | Interleukin 15 | |  |  |  |  |  |
| ENSG00000164308 | | ERAP2 | Endoplasmic reticulum aminopeptidase 2 | | |  |  |  |  |
| ENSG00000165272 | | AQP3 | Aquaporin 3 |  |  |  |  |  |  |
| ENSG00000165806 | | CASP7 | Caspase 7 |  |  |  |  |  |  |
| ENSG00000165895 | | ARHGAP42 | Rho GTPase activating protein 42 | | |  |  |  |  |
| ENSG00000166592 | | RRAD | Ras-related associated with diabetes | | |  |  |  |  |
| ENSG00000166710 | | B2M | Beta-2-microglobulin | |  |  |  |  |  |
| ENSG00000166863 | | TAC3 | Tachykinin precursor 3 | |  |  |  |  |  |
| ENSG00000167550 | | RHEBL1 | Ras homolog enriched in brain like 1 | | |  |  |  |  |
| ENSG00000167995 | | BEST1 | Bestrophin 1 |  |  |  |  |  |  |
| ENSG00000168065 | | SLC22A11 | Solute carrier family 22 member 11 | | |  |  |  |  |
| ENSG00000168404 | | MLKL | Mixed lineage kinase domain like pseudokinase | | | |  |  |  |
| ENSG00000169429 | | CXCL8 | C-X-C motif chemokine ligand 8 (IL-8) | | |  |  |  |  |
| ENSG00000170703 | | TTLL6 | Tubulin tyrosine ligase like 6 | | |  |  |  |  |
| ENSG00000170909 | | OSCAR | Osteoclast associated immunoglobulin-like receptor | | | |  |  |  |
| ENSG00000172602 | | RND1 | Rho family GTPase 1 | |  |  |  |  |  |
| ENSG00000172936 | | MYD88 | Myeloid differentiation primary response 88 | | | |  |  |  |
| ENSG00000174564 | | IL20RB | Interleukin 20 receptor subunit beta | | |  |  |  |  |
| ENSG00000175197 | | DDIT3 | DNA damage inducible transcript 3 | | |  |  |  |  |
| ENSG00000176046 | | NUPR1 | Nuclear protein 1, transcriptional regulator | | | |  |  |  |
| ENSG00000179344 | | HLA-DQB1 | Major histocompatibility complex, class II, DQ beta 1 | | | |  |  |  |
| ENSG00000179431 | | FJX1 | Four jointed box 1 | |  |  |  |  |  |
| ENSG00000180535 | | BHLHA15 | Basic helix-loop-helix family member A15 | | |  |  |  |  |
| ENSG00000182179 | | UBA7 | Ubiquitin-like modifier activating enzyme 7 | | | |  |  |  |
| ENSG00000182393 | | IFNL1 | Interferon lambda 1 | |  |  |  |  |  |
| ENSG00000182782 | | HCAR2 | Hydroxycarboxylic acid receptor 2 | | |  |  |  |  |
| ENSG00000183889 | | NPIPA9 | Nuclear pore complex interacting protein family member A9 | | | | |  |  |
| ENSG00000184371 | | CSF1 | Colony stimulating factor 1 | |  |  |  |  |  |
| ENSG00000184898 | | RBM43 | RNA binding motif protein 43 | | |  |  |  |  |
| ENSG00000185338 | | SOCS1 | Suppressor of cytokine signaling 1 | | |  |  |  |  |
| ENSG00000185880 | | TRIM69 | Tripartite motif containing 69 | | |  |  |  |  |
| ENSG00000196116 | | TDRD7 | Tudor domain containing 7 | |  |  |  |  |  |
| ENSG00000196517 | | SLC6A9 | Solute carrier family 6 member 9 | | |  |  |  |  |
| ENSG00000203650 | | LINC01285 | Long intergenic non-coding RNA 1285 | | |  |  |  |  |
| ENSG00000203697 | | CAPN8 | Calpain 8 |  |  |  |  |  |  |
| ENSG00000204525 | | HLA-C | Major histocompatibility complex, class I, C | | | |  |  |  |
| ENSG00000204542 | | C6orf15 | Chromosome 6 open reading frame 15 | | |  |  |  |  |
| ENSG00000204592 | | HLA-E | Major histocompatibility complex, class I, E | | | |  |  |  |
| ENSG00000204616 | | TRIM31 | Tripartite motif containing 31 | | |  |  |  |  |
| ENSG00000205038 | | PKHD1L1 | Polycystic kidney and hepatic disease 1 like 1 | | | |  |  |  |
| ENSG00000206337 | | HCP5 | HLA complex P5 | |  |  |  |  |  |
| ENSG00000212195 | | U3 | U3 small nucleolar ribonucleoprotein | | |  |  |  |  |
| ENSG00000213886 | | UBD | Ubiquitin D |  |  |  |  |  |  |
| ENSG00000223511 | | LINC02968 | Long intergenic non-coding RNA 2968 | | |  |  |  |  |
| ENSG00000227619 | | ENSG00000227619 | [No HGNC name; likely a novel lncRNA or pseudogene] | | | |  |  |  |
| ENSG00000229056 | | HECW2-AS1 | HECW2 antisense RNA 1 | |  |  |  |  |  |
| ENSG00000229278 | | ENSG00000229278 | [Uncharacterized; Ensembl ID] | | |  |  |  |  |
| ENSG00000229656 | | ITGB1-DT | ITGB1 divergent transcript | |  |  |  |  |  |
| ENSG00000230647 | | ENSG00000230647 | [Uncharacterized; Ensembl ID] | | |  |  |  |  |
| ENSG00000230882 | | ENSG00000230882 | [Uncharacterized; Ensembl ID] | | |  |  |  |  |
| ENSG00000235899 | | LINC01564 | Long intergenic non-coding RNA 1564 | | |  |  |  |  |
| ENSG00000236404 | | VLDLR-AS1 | VLDLR antisense RNA 1 | |  |  |  |  |  |
| ENSG00000237289 | | CKMT1B | Creatine kinase, mitochondrial 1B | | |  |  |  |  |
| ENSG00000240476 | | LINC00973 | Long intergenic non-coding RNA 973 | | |  |  |  |  |
| ENSG00000245648 | | KLRK1-AS1 | KLRK1 antisense RNA 1 | |  |  |  |  |  |
| ENSG00000250508 | | LINC02701 | Long intergenic non-coding RNA 2701 | | |  |  |  |  |
| ENSG00000253616 | | CHILL1 | [Possibly "Chitinase-like 1", but name unclear] | | | |  |  |  |
| ENSG00000254233 | | LINC02365 | Long intergenic non-coding RNA 2365 | | |  |  |  |  |
| ENSG00000259070 | | LINC00639 | Long intergenic non-coding RNA 639 | | |  |  |  |  |
| ENSG00000261618 | | LINC02605 | Long intergenic non-coding RNA 2605 | | |  |  |  |  |
| ENSG00000263563 | | UBBP4 | Ubiquitin B pseudogene 4 | |  |  |  |  |  |
| ENSG00000266947 | | ENSG00000266947 | [Uncharacterized; Ensembl ID] | | |  |  |  |  |
| ENSG00000267060 | | PTGES3L | Prostaglandin E synthase 3 like | | |  |  |  |  |
| ENSG00000272555 | | ENSG00000272555 | [Uncharacterized; Ensembl ID] | | |  |  |  |  |
| ENSG00000272669 | | ENSG00000272669 | [Uncharacterized; Ensembl ID] | | |  |  |  |  |
| ENSG00000273338 | | ENSG00000273338 | [Uncharacterized; Ensembl ID] | | |  |  |  |  |
| ENSG00000273703 | | H2BC14 | H2B clustered histone 14 | |  |  |  |  |  |
| ENSG00000276710 | | CSPG4P10 | Chondroitin sulfate proteoglycan 4 pseudogene 10 | | | |  |  |  |
| ENSG00000276850 | | ENSG00000276850 | [Uncharacterized; Ensembl ID] | | |  |  |  |  |
| ENSG00000280205 | | ENSG00000280205 | [Uncharacterized; Ensembl ID] | | |  |  |  |  |
| ENSG00000286176 | | ENSG00000286176 | [Uncharacterized; Ensembl ID] | | |  |  |  |  |
| ENSG00000287064 | | ENSG00000287064 | [Uncharacterized; Ensembl ID] | | |  |  |  |  |
| ENSG00000287689 | | ENSG00000287689 | [Uncharacterized; Ensembl ID] | | |  |  |  |  |
| ENSG00000287839 | | ENSG00000287839 | [Uncharacterized; Ensembl ID] | | |  |  |  |  |
| ENSG00000288587 | | ENSG00000288587 | [Uncharacterized; Ensembl ID] | | |  |  |  |  |
| ENSG00000289928 | | ENSG00000289928 | [Uncharacterized; Ensembl ID] | | |  |  |  |  |
| ENSG00000290074 | | ENSG00000290074 | [Uncharacterized; Ensembl ID] | | |  |  |  |  |
| ENSG00000290122 | | ENSG00000290122 | [Uncharacterized; Ensembl ID] | | |  |  |  |  |
| ENSG00000290394 | | ENSG00000290394 | [Uncharacterized; Ensembl ID] | | |  |  |  |  |
| ENSG00000290788 | | ENSG00000290788 | [Uncharacterized; Ensembl ID] | | |  |  |  |  |
| ENSG00000291008 | | ENSG00000291008 | [Uncharacterized; Ensembl ID] | | |  |  |  |  |
| ENSG00000291074 | | CCDC144NL | Coiled-coil domain containing 144 family N terminal like | | | |  |  |  |

**Table S6: List of the 12 upregulated genes identified for RPS17, RNF19A and Kernow p6 at 168h post-infection.** This table includes genes that are commonly significantly upregulated for RPS17, RNF19A and Kernow p6. For each gene, the table provides the Ensembl gene ID, the gene symbol and the complete name.

| GENE ID |  | GENE SYMBOL | | GENE NAME |  |  |  |
| --- | --- | --- | --- | --- | --- | --- | --- |
| ENSG00000025708 | | TYMP |  | Thymidine Phosphorylase | |  |  |
| ENSG00000079263 | | SP140 |  | SP140 Nuclear Body Protein | | |  |
| ENSG00000105559 | | PLEKHA4 |  | Pleckstrin Homology Domain Containing A4 | | | |
| ENSG00000130303 | | BST2 |  | Bone Marrow Stromal Cell Antigen 2 | | |  |
| ENSG00000140279 | | DUOX2 |  | Dual Oxidase 2 | |  |  |
| ENSG00000145649 | | GZMA |  | Granzyme A |  |  |  |
| ENSG00000148346 | | LCN2 |  | Lipocalin 2 |  |  |  |
| ENSG00000164236 | | ANKRD33B |  | Ankyrin Repeat Domain 33B | | |  |
| ENSG00000174808 | | BTC |  | Betacellulin |  |  |  |
| ENSG00000250264 | | ENSG00000250264 | | Novel protein, TAP2–HLA-DOB readthrough | | | |
| ENSG00000275302 | | CCL4 |  | C-C Motif Chemokine Ligand 4 | | |  |
| ENSG00000291144 | | ENSG00000291144 | | Uncharacterized / Non-coding transcript | | |  |

**Table S7: List of the 18 upregulated genes identified for ZNF787, RNF19A and Kernow p6 at 168h post-infection.** This table includes genes that are commonly significantly upregulated for ZNF787, RNF19A and Kernow p6. For each gene, the table provides the Ensembl gene ID, the gene symbol and the complete name.

| GENE ID |  | GENE SYMBOL | | GENE NAME |  |  |  |  |
| --- | --- | --- | --- | --- | --- | --- | --- | --- |
| ENSG00000023445 | | BIRC3 |  | Baculoviral IAP Repeat Containing 3 | | |  |  |
| ENSG00000047365 | | ARAP2 |  | ArfGAP With RhoGAP Domain, Ankyrin Repeat And PH Domain 2 | | | | |
| ENSG00000105939 | | ZC3HAV1 |  | Zinc Finger CCCH-Type Containing, Antiviral 1 | | | |  |
| ENSG00000112182 | | BACH2 |  | BTB And CNC Homolog 2 | |  |  |  |
| ENSG00000117226 | | GBP3 |  | Guanylate Binding Protein 3 | | |  |  |
| ENSG00000121060 | | TRIM25 |  | Tripartite Motif Containing 25 | | |  |  |
| ENSG00000123901 | | GPR83 |  | G Protein-Coupled Receptor 83 | | |  |  |
| ENSG00000128567 | | PODXL |  | Podocalyxin |  |  |  |  |
| ENSG00000132256 | | TRIM5 |  | Tripartite Motif Containing 5 | | |  |  |
| ENSG00000152689 | | RASGRP3 |  | RAS Guanyl Releasing Protein 3 | | |  |  |
| ENSG00000164342 | | TLR3 |  | Toll-Like Receptor 3 | |  |  |  |
| ENSG00000179954 | | SSC5D |  | Scavenger Receptor Cysteine Rich Domain Containing 5D | | | | |
| ENSG00000181381 | | DDX60L |  | DEAD-Box Helicase 60 Like | |  |  |  |
| ENSG00000197153 | | H3C12 |  | H3 Histone, Clustered Family Member 12 | | |  |  |
| ENSG00000198133 | | TMEM229B |  | Transmembrane Protein 229B | | |  |  |
| ENSG00000223960 | | CHROMR |  | Chromatin Regulator RNA (lncRNA) | | |  |  |
| ENSG00000289341 | | ENSG00000289341 | | Uncharacterized / Non-coding transcript | | |  |  |
| ENSG00000289720 | | ENSG00000289720 | | Uncharacterized / Non-coding transcript | | |  |  |
|  |  |  |  |  |  |  |  |  |

**Table S8: List of the 8 upregulated genes identified for EEF1A1, RNF19A and Kernow-p6 at 168h post-infection.** For each gene, the table provides the Ensembl gene ID, the gene symbol and the complete name.

| GENE ID |  | GENE SYMBOL | | GENE NAME |  |  |  |
| --- | --- | --- | --- | --- | --- | --- | --- |
| ENSG00000105855 | | ITGB8 |  | Integrin Subunit Beta 8 | | | |
| ENSG00000110944 | | IL23A | | Interleukin 23 Subunit Alpha | | |  |
| ENSG00000116701 | | NCF2 |  | Neutrophil Cytosolic Factor 2 | |  |  |
| ENSG00000120937 | | NPPB |  | Natriuretic Peptide B | | |  |
| ENSG00000137821 | | LRRC49 |  | Leucine Rich Repeat Containing 49 | |  |  |
| ENSG00000147883 | | CDKN2B | | Cyclin Dependent Kinase Inhibitor 2B | | |  |
| ENSG00000171132 | | PRKCE |  | Protein Kinase C Epsilon | |  |  |
| ENSG00000223638 | | RFPL4A | | Ret Finger Protein Like 4A | | |  |

**Table S9: List of the 25 upregulated genes identified for the VHRVs with a replicative advantage at 168h post-infection.** This table includes genes that are commonly significantly upregulated for EEF1A1, KIF1B, RPS17, ZNF787, RNF19A and Kernow p6. For each gene, the table provides the Ensembl gene ID, the gene symbol and the complete name.

| GENE ID |  | GENE SYMBOL | GENE NAME |  |  |  |  |
| --- | --- | --- | --- | --- | --- | --- | --- |
| ENSG00000089127 | | OAS1 | 2′-5′-Oligoadenylate Synthetase 1 | | |  |  |
| ENSG00000111335 | | OAS2 | 2′-5′-Oligoadenylate Synthetase 2 | | |  |  |
| ENSG00000119917 | | IFIT3 | Interferon-Induced Protein with Tetratricopeptide Repeats 3 | | | | |
| ENSG00000119922 | | IFIT2 | Interferon-Induced Protein with Tetratricopeptide Repeats 2 | | | | |
| ENSG00000126709 | | IFI6 | Interferon Alpha Inducible Protein 6 | | |  |  |
| ENSG00000128422 | | KRT17 | Keratin 17 |  |  |  |  |
| ENSG00000130775 | | THEMIS2 | Thymocyte Selection Associated 2 | | |  |  |
| ENSG00000133250 | | ZNF414 | Zinc Finger Protein 414 | |  |  |  |
| ENSG00000134321 | | RSAD2 | Radical S-Adenosyl Methionine Domain Containing 2 | | | |  |
| ENSG00000135114 | | OASL | 2′-5′-Oligoadenylate Synthetase Like | | |  |  |
| ENSG00000137628 | | DDX60 | DExD/H-Box Helicase 60 | |  |  |  |
| ENSG00000137965 | | IFI44 | Interferon-Induced Protein 44 | | |  |  |
| ENSG00000138646 | | HERC5 | HECT and RLD Domain Containing E3 Ubiquitin Protein Ligase 5 | | | | |
| ENSG00000141682 | | PMAIP1 | Phorbol-12-Myristate-13-Acetate-Induced Protein 1 | | | |  |
| ENSG00000157601 | | MX1 | Myxovirus Resistance 1 | |  |  |  |
| ENSG00000162654 | | GBP4 | Guanylate Binding Protein 4 | | |  |  |
| ENSG00000163121 | | NEURL3 | Neuralized E3 Ubiquitin Protein Ligase 3 | | |  |  |
| ENSG00000165949 | | IFI27 | Interferon Alpha Inducible Protein 27 | | |  |  |
| ENSG00000169245 | | CXCL10 | C-X-C Motif Chemokine Ligand 10 | | |  |  |
| ENSG00000169248 | | CXCL11 | C-X-C Motif Chemokine Ligand 11 | | |  |  |
| ENSG00000174898 | | CATSPERD | Cation Channel Sperm Associated Auxiliary Subunit D | | | |  |
| ENSG00000185745 | | IFIT1 | Interferon-Induced Protein with Tetratricopeptide Repeats 1 | | | | |
| ENSG00000213928 | | IRF9 | Interferon Regulatory Factor 9 | | |  |  |
| ENSG00000256500 | | ENSG00000256500 | Kinesin Light Chain 1 Antisense RNA | | |  |  |
| ENSG00000271503 | | CCL5 | C-C Motif Chemokine Ligand 5 | | |  |  |

**Table S10: List of the 45 upregulated genes identified for 4 of the VHRVs with a replicative advantage at 168h post-infection.** This table includes genes that are commonly significantly upregulated for RPS17, ZNF787, RNF19A and Kernow p6. For each gene, the table provides the Ensembl gene ID, the gene symbol and the complete name.

| GENE ID |  | GENE SYMBOL | | GENE NAME |  |  |  |  |
| --- | --- | --- | --- | --- | --- | --- | --- | --- |
| ENSG00000043355 | | ZIC2 |  | Zinc Finger Protein ZIC2 | |  |  |  |
| ENSG00000055332 | | EIF2AK2 |  | Eukaryotic Translation Initiation Factor 2-Alpha Kinase 2 | | | | |
| ENSG00000059378 | | PARP12 |  | Poly(ADP-Ribose) Polymerase 12 | | |  |  |
| ENSG00000067066 | | SP100 |  | SP100 Nuclear Antigen | |  |  |  |
| ENSG00000068079 | | IFI35 |  | Interferon-Induced Protein 35 | | |  |  |
| ENSG00000106560 | | GIMAP2 |  | GTPase, IMAP Family Member 2 | | |  |  |
| ENSG00000111912 | | NCOA7 |  | Nuclear Receptor Coactivator 7 | | |  |  |
| ENSG00000120217 | | CD274 |  | Programmed Cell Death Ligand 1 (PD-L1) | | |  |  |
| ENSG00000121858 | | TNFSF10 |  | TNF Superfamily Member 10 (TRAIL) | | |  |  |
| ENSG00000124201 | | ZNFX1 |  | Zinc Finger, NFX1-Type Containing 1 | | |  |  |
| ENSG00000126243 | | LRFN3 |  | Leucine Rich Repeat and Fibronectin Type III Domain Containing 3 | | | | |
| ENSG00000130813 | | SHFL |  | Shiftless (Antiviral Inhibitor of Ribosomal Frameshifting) | | | |  |
| ENSG00000132109 | | TRIM21 |  | Tripartite Motif Containing 21 | | |  |  |
| ENSG00000138035 | | PNPT1 |  | Polyribonucleotide Nucleotidyltransferase 1 | | | |  |
| ENSG00000140853 | | NLRC5 |  | NLR Family CARD Domain Containing 5 | | |  |  |
| ENSG00000146859 | | TMEM140 |  | Transmembrane Protein 140 | | |  |  |
| ENSG00000155629 | | PIK3AP1 |  | Phosphoinositide-3-Kinase Adaptor Protein 1 | | | |  |
| ENSG00000156587 | | UBE2L6 |  | Ubiquitin Conjugating Enzyme E2 L6 | | |  |  |
| ENSG00000163840 | | DTX3L |  | Deltex E3 Ubiquitin Ligase 3L | | |  |  |
| ENSG00000166750 | | SLFN5 |  | Schlafen Family Member 5 | |  |  |  |
| ENSG00000168062 | | BATF2 |  | Basic Leucine Zipper ATF-Like Transcription Factor 2 | | | |  |
| ENSG00000168394 | | TAP1 |  | Transporter 1, ATP Binding Cassette Subfamily | | | |  |
| ENSG00000168961 | | LGALS9 |  | Galectin 9 |  |  |  |  |
| ENSG00000173193 | | PARP14 |  | Poly(ADP-Ribose) Polymerase 14 | | |  |  |
| ENSG00000173821 | | RNF213 |  | Ring Finger Protein 213 | |  |  |  |
| ENSG00000177335 | | LINC02904 |  | Long Intergenic Non-Protein Coding RNA 2904 | | | |  |
| ENSG00000178685 | | PARP10 |  | Poly(ADP-Ribose) Polymerase 10 | | |  |  |
| ENSG00000183486 | | MX2 |  | Myxovirus Resistance 2 | |  |  |  |
| ENSG00000184979 | | USP18 |  | Ubiquitin Specific Peptidase 18 | | |  |  |
| ENSG00000196684 | | HSH2D |  | Hematopoietic SH2 Domain-Containing | | |  |  |
| ENSG00000197536 | | IRF1-AS1 |  | Interferon Regulatory Factor 1 Antisense RNA | | | |  |
| ENSG00000203797 | | DDO |  | D-Aspartate Oxidase | |  |  |  |
| ENSG00000204261 | | PSMB8-AS1 |  | Proteasome Subunit Beta 8 Antisense RNA 1 | | | |  |
| ENSG00000204264 | | PSMB8 |  | Proteasome Subunit Beta 8 | |  |  |  |
| ENSG00000204267 | | TAP2 |  | Transporter 2, ATP Binding Cassette Subfamily | | | |  |
| ENSG00000221963 | | APOL6 |  | Apolipoprotein L6 | |  |  |  |
| ENSG00000231852 | | CYP21A2 |  | Cytochrome P450 Family 21 Subfamily A Member 2 | | | |  |
| ENSG00000234745 | | HLA-B |  | Major Histocompatibility Complex, Class I, B | | | |  |
| ENSG00000240065 | | PSMB9 |  | Proteasome Subunit Beta 9 | |  |  |  |
| ENSG00000258659 | | TRIM34 |  | Tripartite Motif Containing 34 | | |  |  |
| ENSG00000269720 | | CCDC194 |  | Coiled-Coil Domain Containing 194 | | |  |  |
| ENSG00000272941 | | ENSG00000272941 | | Novel Transcript Antisense to C7orf49 (lncRNA) | | | |  |
| ENSG00000282851 | | BISPR |  | BST2 Interferon-Stimulated Positive Regulator (lncRNA) | | | |  |
| ENSG00000290727 | | ALMS1P1 |  | Alstrom Syndrome 1 Pseudogene 1 | | |  |  |
| ENSG00000291077 | | ENSG00000291077 | | Uncharacterized / Non-coding transcript (lncRNA) | | | |  |

**Table S11: List of the 135 downregulated genes identified for Kernow-p6 at 168h post-infection.** This table includes genes that are commonly significantly downregulated for Kernow p6. For each gene, the table provides the Ensembl gene ID, the gene symbol and the complete name.

| GENE ID | GENE SYMBOL | GENE NAME |  |  |
| --- | --- | --- | --- | --- |
| ENSG00000054356 | PTPRN | Protein tyrosine phosphatase receptor type N |  |  |
| ENSG00000055955 | ITIH4 | Inter-alpha-trypsin inhibitor heavy chain 4 |  |  |
| ENSG00000063015 | SEZ6 | Seizure related 6 homolog |  |  |
| ENSG00000091106 | NLRC4 | NLR family CARD domain containing 4 |  |  |
| ENSG00000101336 | HCK | Hemopoietic cell kinase |  |  |
| ENSG00000103742 | IGDCC4 | Immunoglobulin superfamily DCC subclass member 4 |  |  |
| ENSG00000105088 | OLFM2 | Olfactomedin 2 |  |  |
| ENSG00000110881 | ASIC1 | Acid sensing ion channel subunit 1 |  |  |
| ENSG00000112818 | MEP1A | Meprin A subunit alpha |  |  |
| ENSG00000116783 | TNNI3K | TNNI3 interacting kinase |  |  |
| ENSG00000120049 | KCNIP2 | Potassium voltage-gated channel interacting protein 2 |  |  |
| ENSG00000120903 | CHRNA2 | Cholinergic receptor nicotinic alpha 2 subunit |  |  |
| ENSG00000122729 | ACO1 | Aconitase 1 |  |  |
| ENSG00000133256 | PDE6B | Phosphodiesterase 6B, cGMP-specific, rod, beta |  |  |
| ENSG00000137970 | RPL7P9 | Ribosomal protein L7 pseudogene 9 |  |  |
| ENSG00000139874 | SSTR1 | Somatostatin receptor 1 |  |  |
| ENSG00000144152 | FBLN7 | Fibulin 7 |  |  |
| ENSG00000147059 | SPIN2A | Spindlin family member 2A |  |  |
| ENSG00000147437 | GNRH1 | Gonadotropin releasing hormone 1 |  |  |
| ENSG00000148541 | FAM13C | Family with sequence similarity 13 member C |  |  |
| ENSG00000152580 | IGSF10 | Immunoglobulin superfamily member 10 |  |  |
| ENSG00000152931 | PART1 | Prostate androgen-regulated transcript 1 (lncRNA) |  |  |
| ENSG00000154783 | FGD5 | FYVE, RhoGEF and PH domain containing 5 |  |  |
| ENSG00000156515 | HK1 | Hexokinase 1 |  |  |
| ENSG00000158955 | WNT9B | Wnt family member 9B |  |  |
| ENSG00000159167 | STC1 | Stanniocalcin 1 |  |  |
| ENSG00000162461 | SLC25A34 | Solute carrier family 25 member 34 |  |  |
| ENSG00000163141 | BNIPL | BCL2/adenovirus E1B 19kDa interacting protein like |  |  |
| ENSG00000163810 | TGM4 | Transglutaminase 4 |  |  |
| ENSG00000165194 | PCDH19 | Protocadherin 19 |  |  |
| ENSG00000171817 | ZNF540 | Zinc finger protein 540 |  |  |
| ENSG00000178464 | RPL10P16 | Ribosomal protein L10 pseudogene 16 |  |  |
| ENSG00000178878 | APOLD1 | Apolipoprotein L domain containing 1 |  |  |
| ENSG00000182492 | BGN | Biglycan |  |  |
| ENSG00000183604 | SMG1P5 | SMG1 pseudogene 5 |  |  |
| ENSG00000183775 | KCTD16 | Potassium channel tetramerization domain containing 16 |  |  |
| ENSG00000184270 | H2AC21 | H2A clustered histone 21 |  |  |
| ENSG00000185585 | OLFML2A | Olfactomedin like 2A |  |  |
| ENSG00000185641 | ENSG00000185641 | Gene locus (Ensembl ID, nom officiel non assigné clairement) |  |  |
| ENSG00000187951 | ENSG00000187951 | Gene locus (Ensembl ID, nom officiel non assigné clairement) |  |  |
| ENSG00000196220 | SRGAP3 | SLIT-ROBO Rho GTPase-activating protein 3 |  |  |
| ENSG00000200913 | SNORD46 | Small nucleolar RNA, C/D box 46 |  |  |
| ENSG00000203867 | RBM20 | RNA binding motif protein 20 |  |  |
| ENSG00000203942 | C10orf62 | Chromosome 10 open reading frame 62 |  |  |
| ENSG00000208772 | SNORD94 | Small nucleolar RNA, C/D box 94 |  |  |
| ENSG00000209582 | SNORA48 | Small nucleolar RNA, H/ACA box 48 |  |  |
| ENSG00000212283 | SNORD89 | Small nucleolar RNA, C/D box 89 |  |  |
| ENSG00000213326 | RPS7P11 | Ribosomal protein S7 pseudogene 11 |  |  |
| ENSG00000213366 | GSTM2 | Glutathione S-transferase mu 2 |  |  |
| ENSG00000213553 | RPLP0P6 | Ribosomal protein lateral stalk subunit P0 pseudogene 6 |  |  |
| ENSG00000213759 | UGT2B11 | UDP Glucuronosyltransferase Family 2 Member B11 |  |  |
| ENSG00000213862 | RPL7AP62 | Ribosomal Protein L7 Pseudogene 62 |  |  |
| ENSG00000214784 | RPS3AP21 | Ribosomal Protein S3A Pseudogene 21 |  |  |
| ENSG00000218227 | ENSG00000218227 | Uncharacterized LOC100288637 (pseudogene) |  |  |
| ENSG00000220793 | RPL21P119 | Ribosomal Protein L21 Pseudogene 119 |  |  |
| ENSG00000222328 | RNU2-2P | RNA, U2 small nuclear 2, pseudogene |  |  |
| ENSG00000224858 | RPL29P11 | Ribosomal Protein L29 Pseudogene 11 |  |  |
| ENSG00000225200 | ENSG00000225200 | Uncharacterized LOC100288 |  |  |
| ENSG00000226360 | RPL10AP6 | Ribosomal Protein L7 Pseudogene 22 |  |  |
| ENSG00000226525 | RPS7P10 | Caspase 16, pseudogene |  |  |
| ENSG00000227331 | RPL7AP22 | Ribosomal Protein L7 Pseudogene 22 |  |  |
| ENSG00000228146 | CASP16P | Caspase 16, pseudogene |  |  |
| ENSG00000228205 | RPS3P3 | Ribosomal Protein S3 Pseudogene 3 |  |  |
| ENSG00000228232 | GAPDHP1 | Glyceraldehyde-3-Phosphate Dehydrogenase Pseudogene 1 |  |  |
| ENSG00000228835 | ENSG00000228835 | Uncharacterized LOC100288637 (pseudogene) |  |  |
| ENSG00000230432 | ENSG00000230432 | Uncharacterized LOC100288637 (pseudogene) |  |  |
| ENSG00000231249 | ITPR1-DT | ITPR1 Divergent Transcript (lncRNA) |  |  |
| ENSG00000233293 | ENSG00000233293 | Uncharacterized LOC100288637 (pseudogene) |  |  |
| ENSG00000233392 | UICLM | Uncharacterized lncRNA (UICLM) |  |  |
| ENSG00000233435 | AGGF1P2 | Angiogenic Factor with G-patch and FHA domains 1, pseudogene 2 |  |  |
| ENSG00000233913 | RPL10P9 | Ribosomal Protein L10 Pseudogene 9 |  |  |
| ENSG00000235933 | LINC01779 | Long Intergenic Non-Protein Coding RNA 1779 |  |  |
| ENSG00000236078 | LINC01447 | Long Intergenic Non-Protein Coding RNA 1447 |  |  |
| ENSG00000240376 | ENSG00000240376 | Uncharacterized LOC100288637 (pseudogene) |  |  |
| ENSG00000242071 | RPL7AP6 | Ribosomal Protein L7 Pseudogene 6 |  |  |
| ENSG00000243071 | RPL21P98 | Ribosomal Protein L21 Pseudogene 98 |  |  |
| ENSG00000243199 | RPL6P10 | Ribosomal Protein L6 Pseudogene 10 |  |  |
| ENSG00000244313 | ENSG00000244313 | Uncharacterized LOC100288637 (pseudogene) |  |  |
| ENSG00000244480 | ENSG00000244480 | Uncharacterized LOC100288637 (pseudogene) |  |  |
| ENSG00000245468 | LINC02447 | Long Intergenic Non-Protein Coding RNA 2447 |  |  |
| ENSG00000246877 | DNM1P35 | Dynamin 1 pseudogene 35 |  |  |
| ENSG00000248858 | FLJ46284 | Uncharacterized protein FLJ46284 |  |  |
| ENSG00000249669 | CARMN | Cardiac mesoderm enhancer-associated non-coding RNA |  |  |
| ENSG00000251259 | ENSG00000251259 | Uncharacterized LOC100288637 (pseudogene) |  |  |
| ENSG00000251323 | LINC02728 | Long Intergenic Non-Protein Coding RNA 2728 |  |  |
| ENSG00000251615 | ENSG00000251615 | Uncharacterized LOC100288637 (pseudogene) |  |  |
| ENSG00000255760 | LINC02422 | Long Intergenic Non-Protein Coding RNA 2422 |  |  |
| ENSG00000258555 | SPECC1L-ADORA2A | SPECC1L-Adora2a readthrough transcript |  |  |
| ENSG00000259316 | ENSG00000259316 | Uncharacterized LOC100288637 (pseudogene) |  |  |
| ENSG00000259326 | ENSG00000259326 | Uncharacterized LOC100288637 (pseudogene) |  |  |
| ENSG00000259347 | ENSG00000259347 | Uncharacterized LOC100288637 (pseudogene) |  |  |
| ENSG00000259623 | ENSG00000259623 | Uncharacterized LOC100288637 (pseudogene) |  |  |
| ENSG00000259994 | ENSG00000259994 | Uncharacterized LOC100288637 (pseudogene) |  |  |
| ENSG00000260272 | ENSG00000260272 | Uncharacterized LOC100288637 (pseudogene) |  |  |
| ENSG00000260400 | ENSG00000260400 | Uncharacterized LOC100288637 (pseudogene) |  |  |
| ENSG00000260417 | ENSG00000260417 | Uncharacterized LOC100288637 (pseudogene) |  |  |
| ENSG00000261051 | ENSG00000261051 | Uncharacterized LOC100288637 (pseudogene) |  |  |
| ENSG00000261357 | ENSG00000261357 | Uncharacterized LOC100288637 (pseudogene) |  |  |
| ENSG00000261366 | MANEA-DT | MANEA divergent transcript (lncRNA) |  |  |
| ENSG00000261829 | ENSG00000261829 | Uncharacterized LOC100288637 (pseudogene) |  |  |
| ENSG00000262074 | SNORD3B-2 | Small nucleolar RNA, C/D box 3B-2 |  |  |
| ENSG00000262165 | C17orf114 | Chromosome 17 open reading frame 114 |  |  |
| ENSG00000266767 | ENSG00000266767 | Uncharacterized LOC100288637 (pseudogene) |  |  |
| ENSG00000267152 | ENSG00000267152 | Uncharacterized LOC100288637 (pseudogene) |  |  |
| ENSG00000267280 | TBX2-AS1 | TBX2 antisense RNA 1 (lncRNA) |  |  |
| ENSG00000268061 | NAPA-AS1 | NAPA antisense RNA 1 (lncRNA) |  |  |
| ENSG00000269837 | IPO5P1 | Importin 5 pseudogene 1 |  |  |
| ENSG00000272356 | ENSG00000272356 | Uncharacterized LOC100288637 (pseudogene) |  |  |
| ENSG00000272647 | ENSG00000272647 | Uncharacterized LOC100288637 (pseudogene) |  |  |
| ENSG00000273033 | LINC02035 | Long Intergenic |  |  |
| ENSG00000273184 | ENSG00000273184 | Long Intergenic Non-Protein Coding RNA 3060 |  |  |
| ENSG00000273297 | LINC03060 | Long Intergenic Non-Protein Coding RNA 3060 |  |  |
| ENSG00000274002 | ENSG00000274002 | Uncharacterized LOC100288637 (pseudogene) |  |  |
| ENSG00000275329 | ENSG00000275329 | Uncharacterized LOC100288637 (pseudogene) |  |  |
| ENSG00000275580 | ENSG00000275580 | Uncharacterized LOC100288637 (pseudogene) |  |  |
| ENSG00000276966 | H4C5 | H4 clustered histone 5 |  |  |
| ENSG00000277011 | ENSG00000277011 | Uncharacterized LOC100288637 (pseudogene) |  |  |
| ENSG00000277399 | GPR179 | G protein-coupled receptor 179 |  |  |
| ENSG00000277998 | ENSG00000277998 | Uncharacterized LOC100288637 (pseudogene) |  |  |
| ENSG00000278705 | H4C2 | H4 clustered histone 2 |  |  |
| ENSG00000279221 | ENSG00000279221 | Uncharacterized LOC100288637 (pseudogene) |  |  |
| ENSG00000279423 | ENSG00000279423 | Uncharacterized LOC100288637 (pseudogene) |  |  |
| ENSG00000279608 | ENSG00000279608 | Uncharacterized LOC100288637 (pseudogene) |  |  |
| ENSG00000280106 | ENSG00000280106 | Uncharacterized LOC100288637 (pseudogene) |  |  |
| ENSG00000281026 | N4BP2L2-IT2 | NEDD4 binding protein 2-like 2 intronic transcript 2 (lncRNA) |  |  |
| ENSG00000286796 | ENSG00000286796 | Uncharacterized LOC100288637 (pseudogene) |  |  |
| ENSG00000287110 | ACAD9-DT | ACAD9 divergent transcript (lncRNA) |  |  |
| ENSG00000287133 | ENSG00000287133 | Uncharacterized LOC100288637 (pseudogene) |  |  |
| ENSG00000287315 | ENSG00000287315 | Uncharacterized LOC100288637 (pseudogene) |  |  |
| ENSG00000287978 | ENSG00000287978 | Uncharacterized LOC100288637 (pseudogene) |  |  |
| ENSG00000289102 | ENSG00000289102 | Uncharacterized LOC100288637 (pseudogene) |  |  |
| ENSG00000289419 | ENSG00000289419 | Uncharacterized LOC100288637 (pseudogene) |  |  |
| ENSG00000289520 | ENSG00000289520 | Uncharacterized LOC100288637 (pseudogene) |  |  |
| ENSG00000290541 | CYP4F29P | Cytochrome P450 family 4 subfamily F member 29, pseudogene |  |  |
| ENSG00000291047 | ENSG00000291047 | Uncharacterized LOC100288637 (pseudogene) |  |  |

**Table S12: List of the 118 downregulated genes identified for ZNF787 at 168h post-infection.** This table includes genes that are commonly significantly downregulated for ZNF787. For each gene, the table provides the Ensembl gene ID, the gene symbol and the complete name.

| GENE ID | GENE SYMBOL | GENE NAME |
| --- | --- | --- |
| ENSG00000007541 | PIGQ | Phosphatidylinositol glycan anchor biosynthesis class Q |
| ENSG00000011052 | NME1-NME2 | NME1-NME2 readthrough transcript |
| ENSG00000063176 | SPHK2 | Sphingosine kinase 2 |
| ENSG00000095596 | CYP26A1 | Cytochrome P450 family 26 subfamily A member 1 |
| ENSG00000100399 | CHADL | Chondroadherin-like protein |
| ENSG00000103253 | HAGHL | Hydroxyacylglutathione hydrolase-like |
| ENSG00000103254 | ANTKMT | Ankyrin repeat and lysine methyltransferase |
| ENSG00000103740 | ACSBG1 | Acyl-CoA synthetase bubblegum family member 1 |
| ENSG00000106006 | HOXA6 | Homeobox A6 |
| ENSG00000111424 | VDR | Vitamin D receptor |
| ENSG00000114923 | SLC4A3 | Solute carrier family 4 member 3 |
| ENSG00000115286 | NDUFS7 | NADH:ubiquinone oxidoreductase subunit S7 |
| ENSG00000117425 | PTCH2 | Patched 2 |
| ENSG00000125122 | FBXL9P | F-box and leucine rich repeat protein 9, pseudogene |
| ENSG00000126464 | PRR12 | Proline rich 12 |
| ENSG00000127586 | CHTF18 | Chromosome transmission fidelity factor 18 homolog |
| ENSG00000127743 | IL17B | Interleukin 17B |
| ENSG00000128346 | C22orf23 | Chromosome 22 open reading frame 23 |
| ENSG00000129173 | E2F8 | E2F transcription factor 8 |
| ENSG00000129195 | PIMREG | PICALM interacting mitotic regulator |
| ENSG00000130751 | NPAS1 | Neuronal PAS domain protein 1 |
| ENSG00000131591 | C1orf159 | Chromosome 1 open reading frame 159 |
| ENSG00000132207 | SLX1A | SLX1 structure-specific endonuclease subunit A |
| ENSG00000135722 | FBXL8 | F-box and leucine rich repeat protein 8 |
| ENSG00000139187 | KLRG1 | Killer cell lectin-like receptor subfamily G member 1 |
| ENSG00000140451 | PIF1 | PIF1 5'-to-3' DNA helicase |
| ENSG00000149043 | SYT8 | Synaptotagmin 8 |
| ENSG00000158106 | RHPN1 | Rhophilin Rho GTPase binding protein 1 |
| ENSG00000160298 | C21orf58 | Chromosome 21 open reading frame 58 |
| ENSG00000161618 | ALDH16A1 | Aldehyde dehydrogenase 16 family member A1 |
| ENSG00000161996 | WDR90 | WD repeat domain 90 |
| ENSG00000163576 | EFHB | EF-hand domain family member B |
| ENSG00000164867 | NOS3 | Nitric oxide synthase 3 (endothelial) |
| ENSG00000167774 | ENSG00000167774 | Uncharacterized LOC100288637 (pseudogene) |
| ENSG00000168298 | H1-4 | H1.4 linker histone, cluster member |
| ENSG00000168913 | ENHO | Energy homeostasis-associated protein |
| ENSG00000169583 | CLIC3 | Chloride intracellular channel 3 |
| ENSG00000169696 | ASPSCR1 | Alveolar soft part sarcoma chromosome region, candidate 1 |
| ENSG00000172367 | NHERF4 | Na+/H+ exchange regulatory cofactor 4 |
| ENSG00000173227 | SYT12 | Synaptotagmin 12 |
| ENSG00000174917 | MICOS13 | MICOS complex subunit 13 |
| ENSG00000175344 | CHRNA7 | Cholinergic receptor nicotinic alpha 7 subunit |
| ENSG00000179403 | VWA1 | Von Willebrand factor A domain containing 1 |
| ENSG00000180573 | H2AC6 | H2A clustered histone 6 |
| ENSG00000181029 | TRAPPC5 | Trafficking protein particle complex 5 |
| ENSG00000182327 | GLTPD2 | Glycolipid transfer protein domain containing 2 |
| ENSG00000182685 | BRICD5 | BRIC domain containing 5 |
| ENSG00000182687 | GALR2 | Galanin receptor 2 |
| ENSG00000182809 | CRIP2 | Cysteine-rich protein 2 |
| ENSG00000184260 | H2AC20 | H2A clustered histone 20 |
| ENSG00000184357 | H1-5 | H1.5 linker histone, cluster member |
| ENSG00000185130 | H2BC13 | H2B clustered histone 13 |
| ENSG00000185347 | TEDC1 | Tubulin epsilon and delta complex 1 |
| ENSG00000187860 | CCDC157 | Coiled-coil domain containing 157 |
| ENSG00000188566 | NDOR1 | NADPH dependent diflavin oxidoreductase 1 |
| ENSG00000196421 | C20orf204 | Chromosome 20 open reading frame 204 |
| ENSG00000196747 | H2AC13 | H2A clustered histone 13 |
| ENSG00000196866 | H2AC7 | H2A clustered histone 7 |
| ENSG00000197180 | ATP6AP1-DT | ATP6AP1 divergent transcript (lncRNA) |
| ENSG00000197182 | MIRLET7BHG | MIRLET7B host gene (lncRNA) |
| ENSG00000197238 | H4C11 | H4 clustered histone 11 |
| ENSG00000197409 | H3C4 | H3 clustered histone 4 |
| ENSG00000197837 | H4C16 | H4 clustered histone 16 |
| ENSG00000198870 | STKLD1 | Serine/threonine kinase like domain containing 1 |
| ENSG00000199631 | SNORD33 | Small nucleolar RNA, C/D box 33 |
| ENSG00000200156 | RNU5B-1 | RNA, U5B small nuclear 1 |
| ENSG00000200783 | RN7SKP180 | RNA, 7SK, pseudogene 180 |
| ENSG00000202512 | RN7SKP230 | RNA, 7SK, pseudogene 230 |
| ENSG00000203811 | H3C14 | H3 clustered histone 14 |
| ENSG00000207008 | SNORA54 | Small nucleolar RNA, H/ACA box 54 |
| ENSG00000207233 | SNORA37 | Small nucleolar RNA, H/ACA box 37 |
| ENSG00000207344 | SNORA22C | Small nucleolar RNA, H/ACA box 22C |
| ENSG00000212464 | SNORA12 | Small nucleolar RNA, H/ACA box 12 |
| ENSG00000213221 | DNLZ | DNL-type zinc finger |
| ENSG00000213347 | MXD3 | MAX dimerization protein 3 |
| ENSG00000215915 | ATAD3C | ATPase family AAA domain-containing protein 3C |
| ENSG00000225285 | LINC01770 | Long Intergenic Non-Protein Coding RNA 1770 |
| ENSG00000225968 | ELFN1 | Extracellular leucine-rich repeat and fibronectin type III domain containing 1 |
| ENSG00000233822 | H2BC15 | H2B clustered histone 15 |
| ENSG00000234327 | ZNF232-AS1 | ZNF232 antisense RNA 1 |
| ENSG00000235169 | SMIM1 | Small integral membrane protein 1 |
| ENSG00000242802 | AP5Z1 | Adaptor related protein complex 5 subunit zeta 1 |
| ENSG00000256092 | SBNO1-AS1 | Strawberry notch homolog 1 antisense RNA 1 |
| ENSG00000258017 | TUBA1B-AS1 | Tubulin alpha 1B antisense RNA 1 |
| ENSG00000258334 | TROAP-AS1 | Trophinin associated protein antisense RNA 1 |
| ENSG00000259112 | NDUFC2-KCTD14 | NDUFC2-KCTD14 readthrough transcript |
| ENSG00000260886 | TAT-AS1 | Tyrosine aminotransferase antisense RNA 1 |
| ENSG00000261732 | ENSG00000261732 | Uncharacterized LOC100288637 (pseudogene) |
| ENSG00000261884 | ENSG00000261884 | Uncharacterized LOC100288637 (pseudogene) |
| ENSG00000262877 | ENSG00000262877 | Uncharacterized LOC100288637 (pseudogene) |
| ENSG00000267001 | ENSG00000267001 | Uncharacterized LOC100288637 (pseudogene) |
| ENSG00000267059 | ENSG00000267059 | Uncharacterized LOC100288637 (pseudogene) |
| ENSG00000270103 | ENSG00000270103 | Uncharacterized LOC100288637 (pseudogene) |
| ENSG00000270276 | H4C15 | H4 clustered histone 15 |
| ENSG00000272661 | ENSG00000272661 | Uncharacterized LOC100288637 (pseudogene) |
| ENSG00000273230 | ENSG00000273230 | Uncharacterized LOC100288637 (pseudogene) |
| ENSG00000273420 | ENSG00000273420 | Uncharacterized LOC100288637 (pseudogene) |
| ENSG00000273542 | H4C12 | H4 clustered histone 12 |
| ENSG00000274600 | RIMBP3B | RIMS binding protein 3B |
| ENSG00000274997 | H2AC12 | H2A clustered histone 12 |
| ENSG00000275713 | H2BC9 | H2B clustered histone 9 |
| ENSG00000276368 | H2AC14 | H2A clustered histone 14 |
| ENSG00000276903 | H2AC16 | H2A clustered histone 16 |
| ENSG00000277027 | RMRP | RNA component of mitochondrial RNA processing endoribonuclease |
| ENSG00000277075 | H2AC8 | H2A clustered histone 8 |
| ENSG00000277775 | H3C7 | H3 clustered histone 7 |
| ENSG00000278249 | SCARNA2 | Small Cajal body-specific RNA 2 |
| ENSG00000278463 | H2AC4 | H2A clustered histone 4 |
| ENSG00000278677 | H2AC17 | H2A clustered histone 17 |
| ENSG00000278828 | H3C10 | H3 clustered histone 10 |
| ENSG00000280496 | SNORA17B | Small nucleolar RNA, H/ACA box 17B |
| ENSG00000286144 | ENSG00000286144 | Uncharacterized LOC100288637 (pseudogene) |
| ENSG00000287080 | H3C3 | H3 clustered histone 3 |
| ENSG00000288859 | H2AC19 | H2A clustered histone 19 |
| ENSG00000288891 | ENSG00000288891 | Uncharacterized LOC100288637 (pseudogene) |
| ENSG00000289532 | ENSG00000289532 | Uncharacterized LOC100288637 (pseudogene) |
| ENSG00000290680 | ENSG00000290680 | Uncharacterized LOC100288637 (pseudogene) |
| ENSG00000290738 | URAHP | Urocanate hydratase pseudogene |

**Table S13: List of the 58 downregulated genes identified for RNF19A at 168h post-infection.** This table includes genes that are commonly significantly downregulated for RNF19A. For each gene, the table provides the Ensembl gene ID, the gene symbol and the complete name.

| GENE ID | GENE SYMBOL | GENE NAME |
| --- | --- | --- |
| ENSG00000106178 | CCL24 | C-C motif chemokine ligand 24 |
| ENSG00000113721 | PDGFRB | Platelet-derived growth factor receptor beta |
| ENSG00000117407 | ARTN | Artemin |
| ENSG00000125508 | SRMS | Src-related kinase lacking C-terminal regulatory tyrosine and N-terminal myristylation sites |
| ENSG00000134376 | CRB1 | Crumbs cell polarity complex component 1 |
| ENSG00000146910 | CNPY1 | Canopy FGF signaling regulator 1 |
| ENSG00000149596 | JPH2 | Junctophilin 2 |
| ENSG00000154358 | OBSCN | Obscurin, cytoskeletal calmodulin and titin-interacting RhoGEF |
| ENSG00000162383 | SLC1A7 | Solute carrier family 1 member 7 |
| ENSG00000165512 | ZNF22 | Zinc finger protein 22 |
| ENSG00000166025 | AMOTL1 | Angiomotin like 1 |
| ENSG00000167117 | ANKRD40CL | Ankyrin repeat domain 40C-like |
| ENSG00000167554 | ZNF610 | Zinc finger protein 610 |
| ENSG00000171388 | APLN | Apelin |
| ENSG00000173530 | TNFRSF10D | TNF receptor superfamily member 10D |
| ENSG00000173546 | CSPG4 | Chondroitin sulfate proteoglycan 4 |
| ENSG00000178440 | TIMM23B-AGAP6 | TIMM23B-AGAP6 readthrough transcript |
| ENSG00000183508 | TENT5C | Terminal nucleotidyltransferase 5C |
| ENSG00000206567 | ENSG00000206567 | Uncharacterized LOC100288637 (pseudogene) |
| ENSG00000212802 | RPL15P3 | Ribosomal protein L15 pseudogene 3 |
| ENSG00000213609 | RPL7AP50 | Ribosomal protein L7 pseudogene 50 |
| ENSG00000213885 | RPL13AP7 | Ribosomal protein L13a pseudogene 7 |
| ENSG00000214353 | VAC14-AS1 | VAC14 antisense RNA 1 |
| ENSG00000214485 | RPL7P1 | Ribosomal protein L7 pseudogene 1 |
| ENSG00000224934 | GOT1-DT | GOT1 divergent transcript (lncRNA) |
| ENSG00000226519 | LINC00390 | Long intergenic non-protein coding RNA 390 |
| ENSG00000227906 | SNAP25-AS1 | SNAP25 antisense RNA 1 |
| ENSG00000227939 | RPL3P2 | Ribosomal protein L3 pseudogene 2 |
| ENSG00000228929 | RPS13P2 | Ribosomal protein S13 pseudogene 2 |
| ENSG00000230629 | RPS23P8 | Ribosomal protein S23 pseudogene 8 |
| ENSG00000231566 | LINC02595 | Long intergenic non-protein coding RNA 2595 |
| ENSG00000232093 | DCST1-AS1 | DCST1 antisense RNA 1 |
| ENSG00000232987 | LINC01219 | Long intergenic non-protein coding RNA 1219 |
| ENSG00000233426 | EIF3FP3 | Eukaryotic translation initiation factor 3 subunit F pseudogene 3 |
| ENSG00000236801 | RPL24P8 | Ribosomal protein L24 pseudogene 8 |
| ENSG00000237017 | PRPF31-AS1 | PRPF31 antisense RNA 1 |
| ENSG00000244363 | RPL7P23 | Ribosomal protein L7 pseudogene 23 |
| ENSG00000250091 | DNAH10OS | Dynein axonemal heavy chain 10 opposite strand |
| ENSG00000250397 | ENSG00000250397 | Uncharacterized LOC100288637 (pseudogene) |
| ENSG00000254206 | NPIPB11 | Nuclear pore complex interacting protein family member B11 |
| ENSG00000254912 | MPHOSPH10P2 | M-phase phosphoprotein 10 pseudogene 2 |
| ENSG00000258768 | ENSG00000258768 | Uncharacterized LOC100288637 (pseudogene) |
| ENSG00000260293 | ENSG00000260293 | Uncharacterized LOC100288637 (pseudogene) |
| ENSG00000264853 | ENSG00000264853 | Uncharacterized LOC100288637 (pseudogene) |
| ENSG00000266076 | ENSG00000266076 | Uncharacterized LOC100288637 (pseudogene) |
| ENSG00000269982 | ENSG00000269982 | Uncharacterized LOC100288637 (pseudogene) |
| ENSG00000271789 | ENSG00000271789 | Uncharacterized LOC100288637 (pseudogene) |
| ENSG00000274750 | H3C6 | H3 clustered histone 6 |
| ENSG00000277182 | ENSG00000277182 | Uncharacterized LOC100288637 (pseudogene) |
| ENSG00000279168 | ENSG00000279168 | Uncharacterized LOC100288637 (pseudogene) |
| ENSG00000285991 | ENSG00000285991 | Uncharacterized LOC100288637 (pseudogene) |
| ENSG00000287502 | ENSG00000287502 | Uncharacterized LOC100288637 (pseudogene) |
| ENSG00000287562 | ENSG00000287562 | Uncharacterized LOC100288637 (pseudogene) |
| ENSG00000287668 | ENSG00000287668 | Uncharacterized LOC100288637 (pseudogene) |
| ENSG00000289475 | ENSG00000289475 | Uncharacterized LOC100288637 (pseudogene) |
| ENSG00000290926 | LINC03006 | Long intergenic non-protein coding RNA 3006 |
| ENSG00000291051 | ENSG00000291051 | Uncharacterized LOC100288637 (pseudogene) |
| ENSG00000291219 | ENSG00000291219 | Uncharacterized LOC100288637 (pseudogene) |

**Table S14: List of the 26 downregulated genes identified for EEF1A1 at 168h post-infection.** This table includes genes that are commonly significantly downregulated for EEF1A1. For each gene, the table provides the Ensembl gene ID, the gene symbol and the complete name.

| GENE ID | GENE SYMBOL | GENE NAME |
| --- | --- | --- |
| ENSG00000009724 | MASP2 | Mannan-binding lectin serine peptidase 2 |
| ENSG00000080573 | COL5A3 | Collagen type V alpha 3 chain |
| ENSG00000081479 | LRP2 | LDL receptor related protein 2 |
| ENSG00000100373 | UPK3A | Uroplakin 3A |
| ENSG00000101746 | NOL4 | Nucleolar protein 4 |
| ENSG00000114113 | RBP2 | Retinol binding protein 2 |
| ENSG00000131482 | G6PC1 | Glucose-6-phosphatase catalytic subunit 1 |
| ENSG00000132437 | DDC | Dopa decarboxylase |
| ENSG00000152475 | ZNF837 | Zinc finger protein 837 |
| ENSG00000165215 | CLDN3 | Claudin 3 |
| ENSG00000168077 | SCARA3 | Scavenger receptor class A member 3 |
| ENSG00000169918 | OTUD7A | OTU deubiquitinase 7A |
| ENSG00000174276 | ZNHIT2 | Zinc finger HIT-type containing 2 |
| ENSG00000177599 | ZNF491 | Zinc finger protein 491 |
| ENSG00000196167 | COLCA1 | Colorectal cancer associated 1 |
| ENSG00000198948 | MFAP3L | Microfibril associated protein 3-like |
| ENSG00000204653 | ASPDH | Aspartate dehydrogenase domain containing |
| ENSG00000213996 | TM6SF2 | Transmembrane 6 superfamily member 2 |
| ENSG00000235374 | SSR4P1 | Signal sequence receptor subunit 4 pseudogene 1 |
| ENSG00000259673 | IQCH-AS1 | IQ motif containing H antisense RNA 1 |
| ENSG00000264538 | SUZ12P1 | SUZ12 pseudogene 1 |
| ENSG00000266402 | SNHG25 | Small nucleolar RNA host gene 25 |
| ENSG00000270722 | RNVU1-31 | RNA, U1 small nuclear 31 |
| ENSG00000285417 | ENSG00000285417 | Uncharacterized LOC100288637 (pseudogene) |
| ENSG00000288864 | ARHGAP11A-SCG5 | ARHGAP11A-SCG5 readthrough transcript |
| ENSG00000289445 | ENSG00000289445 | Uncharacterized LOC100288637 (pseudogene) |

**Table S15: List of the 69 downregulated genes identified for RNF19A and Kernow-p6 at 168h post-infection.** This table includes genes that are commonly significantly downregulated for RNF19A and Kernow-p6. For each gene, the table provides the Ensembl gene ID, the gene symbol and the complete name.

| GENE ID | GENE SYMBOL | GENE NAME |
| --- | --- | --- |
| ENSG00000055957 | ITIH1 | Inter-alpha-trypsin inhibitor heavy chain 1 |
| ENSG00000063046 | EIF4B | Eukaryotic translation initiation factor 4B |
| ENSG00000067992 | PDK3 | Pyruvate dehydrogenase kinase 3 |
| ENSG00000068489 | PRR11 | Proline rich 11 |
| ENSG00000100129 | EIF3L | Eukaryotic translation initiation factor 3 subunit L |
| ENSG00000122406 | RPL5 | Ribosomal protein L5 |
| ENSG00000124942 | AHNAK | AHNAK nucleoprotein |
| ENSG00000125954 | CHURC1-FNTB | CHURC1-FNTB readthrough transcript |
| ENSG00000140873 | ADAMTS18 | ADAM metallopeptidase with thrombospondin type 1 motif 18 |
| ENSG00000145425 | RPS3A | Ribosomal protein S3A |
| ENSG00000147100 | SLC16A2 | Solute carrier family 16 member 2 |
| ENSG00000152954 | NRSN1 | Neurensin 1 |
| ENSG00000153446 | C16orf89 | Chromosome 16 open reading frame 89 |
| ENSG00000156508 | EEF1A1 | Eukaryotic translation elongation factor 1 alpha 1 |
| ENSG00000166033 | HTRA1 | HtrA serine peptidase 1 |
| ENSG00000174444 | RPL4 | Ribosomal protein L4 |
| ENSG00000175886 | RPL7AP66 | Ribosomal protein L7 pseudogene 66 |
| ENSG00000179256 | SMCO3 | Single-pass membrane protein with coiled-coil domain 3 |
| ENSG00000180758 | GPR157 | G protein-coupled receptor 157 |
| ENSG00000181378 | CFAP65 | Cilia and flagella associated protein 65 |
| ENSG00000182752 | PAPPA | Pappalysin 1 |
| ENSG00000183723 | CMTM4 | CKLF like MARVEL transmembrane domain containing 4 |
| ENSG00000196205 | EEF1A1P5 | Eukaryotic translation elongation factor 1 alpha 1 pseudogene 5 |
| ENSG00000197258 | EIF4BP6 | Eukaryotic translation initiation factor 4E binding protein 6 |
| ENSG00000201229 | SNORA63D | Small nucleolar RNA, H/ACA box 63D |
| ENSG00000205871 | RPS3AP47 | Ribosomal protein S3A pseudogene 47 |
| ENSG00000205978 | NYNRIN | NYN domain and retroviral integrase containing |
| ENSG00000207405 | SNORA64 | Small nucleolar RNA, H/ACA box 64 |
| ENSG00000212789 | ST13P5 | ST13 pseudogene 5 |
| ENSG00000214199 | EEF1A1P12 | Eukaryotic translation elongation factor 1 alpha 1 pseudogene 12 |
| ENSG00000214389 | RPS3AP26 | Ribosomal protein S3A pseudogene 26 |
| ENSG00000215244 | LINC02649 | Long intergenic non-protein coding RNA 2649 |
| ENSG00000224307 | LINC02975 | Long intergenic non-protein coding RNA 2975 |
| ENSG00000224546 | EIF4BP3 | Eukaryotic translation initiation factor 4E binding protein 3 |
| ENSG00000225031 | EIF4BP7 | Eukaryotic translation initiation factor 4E binding protein 7 |
| ENSG00000226239 | ENSG00000226239 | Uncharacterized LOC100288637 (pseudogene) |
| ENSG00000229638 | RPL4P4 | Ribosomal protein L4 pseudogene 4 |
| ENSG00000230207 | RPL4P5 | Ribosomal protein L4 pseudogene 5 |
| ENSG00000232472 | EEF1B2P3 | Eukaryotic translation elongation factor 1 beta 2 pseudogene 3 |
| ENSG00000233476 | EEF1A1P6 | Eukaryotic translation elongation factor 1 alpha 1 pseudogene 6 |
| ENSG00000234797 | RPS3AP6 | Ribosomal protein S3A pseudogene 6 |
| ENSG00000235552 | RPL6P27 | Ribosomal protein L6 pseudogene 27 |
| ENSG00000236901 | MIR600HG | MIR600 host gene (lncRNA) |
| ENSG00000249264 | EEF1A1P9 | Eukaryotic translation elongation factor 1 alpha 1 pseudogene 9 |
| ENSG00000249855 | EEF1A1P19 | Eukaryotic translation elongation factor 1 alpha 1 pseudogene 19 |
| ENSG00000250182 | EEF1A1P13 | Eukaryotic translation elongation factor 1 alpha 1 pseudogene 13 |
| ENSG00000254772 | EEF1G | Eukaryotic translation elongation factor 1 gamma |
| ENSG00000259291 | ZNF710-AS1 | ZNF710 antisense RNA 1 |
| ENSG00000259976 | ENSG00000259976 | Uncharacterized LOC100288637 (pseudogene) |
| ENSG00000260804 | LINC01963 | Long intergenic non-protein coding RNA 1963 |
| ENSG00000261087 | ZNNT1 | Zinc finger N-terminal domain containing 1 |
| ENSG00000261996 | ENSG00000261996 | Uncharacterized LOC100288637 (pseudogene) |
| ENSG00000263069 | RNF213-AS1 | RNF213 antisense RNA 1 |
| ENSG00000263731 | ENSG00000263731 | Uncharacterized LOC100288637 (pseudogene) |
| ENSG00000264895 | ENSG00000264895 | Uncharacterized LOC100288637 (pseudogene) |
| ENSG00000269947 | ENSG00000269947 | Uncharacterized LOC100288637 (pseudogene) |
| ENSG00000272942 | ENSG00000272942 | Uncharacterized LOC100288637 (pseudogene) |
| ENSG00000273032 | DGCR5 | DiGeorge syndrome critical region gene 5 (lncRNA) |
| ENSG00000274070 | CASTOR2 | Cellular arginine sensor for mTORC1 subunit 2 |
| ENSG00000275549 | STPG3-AS1 | STPG3 antisense RNA 1 |
| ENSG00000275994 | SNORA24 | Small nucleolar RNA, H/ACA box 24 |
| ENSG00000278873 | PRO1804 | Uncharacterized protein PRO1804 |
| ENSG00000278962 | ENSG00000278962 | Uncharacterized LOC100288637 (pseudogene) |
| ENSG00000279192 | PWAR5 | Prader-Willi region antisense RNA 5 |
| ENSG00000283041 | ENSG00000283041 | Uncharacterized LOC100288637 (pseudogene) |
| ENSG00000285625 | ENSG00000285625 | Uncharacterized LOC100288637 (pseudogene) |
| ENSG00000285629 | ENSG00000285629 | Uncharacterized LOC100288637 (pseudogene) |
| ENSG00000290469 | ENSG00000290469 | Uncharacterized LOC100288637 (pseudogene) |
| ENSG00000290920 | ENSG00000290920 | Uncharacterized LOC100288637 (pseudogene) |

**Table S16: List of the 16 downregulated genes identified for EEF1A1 and ZNF787 at 168h post-infection**

This table includes genes that are commonly significantly downregulated for EEF1A1 and ZNF787 and Kernow-p6. For each gene, the table provides the Ensembl gene ID, the gene symbol and the complete name.

| GENE ID | GENE SYMBOL | GENE NAME |
| --- | --- | --- |
| ENSG00000086506 | HBQ1 | Hemoglobin subunit theta 1 |
| ENSG00000103260 | METRN | Meteorin, glial cell differentiation regulator |
| ENSG00000130881 | LRP3 | LDL receptor related protein 3 |
| ENSG00000145217 | SLC26A1 | Solute carrier family 26-member 1 |
| ENSG00000166126 | AMN | Amnion associated transmembrane protein |
| ENSG00000178814 | OPLAH | 5-oxoprolinase, ATP-hydrolyzing |
| ENSG00000178934 | LGALS7B | Galectin 7B |
| ENSG00000183598 | H3C13 | H3 clustered histone 13 |
| ENSG00000184986 | TMEM121 | Transmembrane protein 121 |
| ENSG00000185838 | GNB1L | G protein subunit beta 1-like |
| ENSG00000199347 | RNU5E-1 | RNA, U5E small nuclear 1 |
| ENSG00000201512 | SNORA71C | Small nucleolar RNA, H/ACA box 71C |
| ENSG00000201558 | RNVU1-6 | RNA, U1 small nuclear 6 |
| ENSG00000203852 | H3C15 | H3 clustered histone 15 |
| ENSG00000206811 | SNORA10 | Small nucleolar RNA, H/ACA box 10 |
| ENSG00000273544 | SNORA44 | Small nucleolar RNA, H/ACA box 44 |
